# Dynamics of miRNA accumulation during *C. elegans* larval development

**DOI:** 10.1093/nar/gkae115

**Published:** 2024-02-21

**Authors:** Smita Nahar, Lucas J Morales Moya, Jana Brunner, Gert-Jan Hendriks, Benjamin Towbin, Yannick P Hauser, Giovanna Brancati, Dimos Gaidatzis, Helge Großhans

**Affiliations:** Friedrich Miescher Institute for Biomedical Research (FMI), Basel, Switzerland; Friedrich Miescher Institute for Biomedical Research (FMI), Basel, Switzerland; Friedrich Miescher Institute for Biomedical Research (FMI), Basel, Switzerland; University of Basel, Basel, Switzerland; Friedrich Miescher Institute for Biomedical Research (FMI), Basel, Switzerland; Friedrich Miescher Institute for Biomedical Research (FMI), Basel, Switzerland; University of Bern, Bern, Switzerland; Friedrich Miescher Institute for Biomedical Research (FMI), Basel, Switzerland; University of Basel, Basel, Switzerland; Friedrich Miescher Institute for Biomedical Research (FMI), Basel, Switzerland; University of Basel, Basel, Switzerland; Friedrich Miescher Institute for Biomedical Research (FMI), Basel, Switzerland; SIB Swiss Institute of Bioinformatics, Basel, Switzerland; Friedrich Miescher Institute for Biomedical Research (FMI), Basel, Switzerland; University of Basel, Basel, Switzerland

## Abstract

Temporally and spatially controlled accumulation underlies the functions of microRNAs (miRNAs) in various developmental processes. In *Caenorhabditis elegans*, this is exemplified by the temporal patterning miRNAs *lin-4* and *let-7*, but for most miRNAs, developmental expression patterns remain poorly resolved. Indeed, experimentally observed long half-lives may constrain possible dynamics. Here, we profile miRNA expression throughout *C. elegans* postembryonic development at high temporal resolution, which identifies dynamically expressed miRNAs. We use mathematical models to explore the underlying mechanisms. For *let-7*, we can explain, and experimentally confirm, a striking stepwise accumulation pattern through a combination of rhythmic transcription and stage-specific regulation of precursor processing by the RNA-binding protein LIN-28. By contrast, the dynamics of several other miRNAs cannot be explained by regulation of production rates alone. Specifically, we show that a combination of oscillatory transcription and rhythmic decay drive rhythmic accumulation of miR-235, orthologous to miR-92 in other animals. We demonstrate that decay of miR-235 and additional miRNAs depends on EBAX-1, previously implicated in target-directed miRNA degradation (TDMD). Taken together, our results provide insight into dynamic miRNA decay and establish a resource to studying both the developmental functions of, and the regulatory mechanisms acting on, miRNAs.

## Graphical Abstract

**Graphical Abstract:**
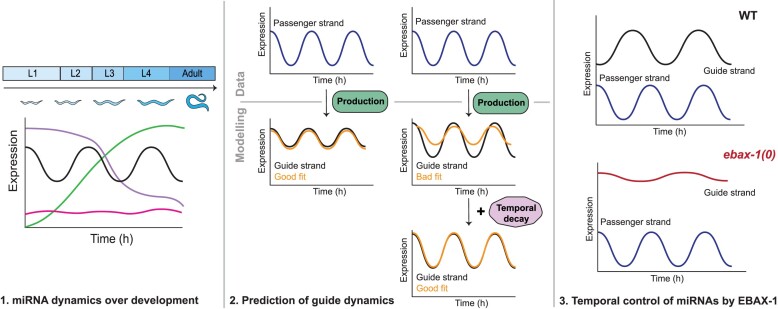

## Introduction

Successful development of a multicellular organism requires cells to adopt specific fates at the right time and in coordination with other cells. This necessitates dynamic, spatiotemporally well-controlled gene expression. In part, such dynamics may be achieved through post-transcriptional regulation by miRNAs, small non-coding RNAs that bind to partially complementary sites on mRNAs to promote transcript degradation or translational repression (1,2). Thus, the iconic *lin-4* and *let-7* miRNAs in *Caenorhabditis elegans* accumulate during the first (L1) or fourth (L4) larval stage to control transition to later larval and adult cell fates, respectively (3,4), by repressing their cognate targets (5,6).

Studies using GFP reporter genes driven by miRNA promoters revealed dynamic transcription of several additional miRNAs in *C. elegans* (7). However, as mature miRNA expression has thus far been examined at low temporal resolution, with sampling typically occurring once per larval stage (8–14), it is unclear to what extent this translates into dynamic accumulation of mature miRNAs. Specifically, long half-lives of mature miRNAs may buffer their levels against the effects of dynamic transcription. Thus, in a striking example from mouse, pri-miR-122 levels were shown to fluctuate in a ∼24-h (circadian) cycle in the liver due to rhythmic transcription, but a long half-life of mature miR-122 led to essentially invariant levels of miR-122 over the same cycle (15).

In *C. elegans*, where a larval stage takes ∼7–10 h and the entire generation time is <3 days (at 25°C), the median half-life of miRNAs was determined to be ∼21 h (16). This and other studies of *C. elegans* miRNA half-lives involved acute perturbations of miRNA production, either by blocking transcription (17) or pri-miRNA processing (16,18), making them susceptible to compensatory or indirect effects on miRNA degradation. Nonetheless, the reported half-lives are in a similar range to those observed by metabolic labeling in mouse and fly cells grown *in vitro* (19,20). Collectively, these results would thus imply limited miRNA dynamics during developmentally relevant timescales.

Since previous experiments to determine miRNA half-lives in *C. elegans* queried only two developmental stages (larval stage L1 and gravid adults), it remains possible that modulated miRNA stability may increase the dynamics of individual miRNAs at specific times. Indeed, miRNAs of the miR-35 family (i.e. miR-35∼42, henceforth *miR-35fam*) were shown to be rapidly cleared during the embryo-to-L1 transition (21). This process relies on EBAX-1/ZSWIM8, a substrate adapter of a Cullin-RING E3 ubiquitin ligase (CRL) complex previously shown to mediate miRNA degradation by inducing proteolytic decay of the protective AGO protein (22,23).

EBAX-1 may target miRNAs more broadly, and additional miRNAs were shown to be upregulated in *ebax-1* mutant relative to wild-type gravid adults (23). However, it is not understood whether destabilization by EBAX-1 is constitutive or developmentally regulated, and the mechanism by which EBAX-1 targets specific miRNAs has remained unclear. In mammals, extensive base pairing (involving both 5′ and 3′ ends) between a miRNA and a ‘trigger’ RNA mediates a conformational change in AGO that promotes AGO binding and/or ubiquitylation by CRL^ZSWIM8^, followed by miRNA degradation. Accordingly, this regulated miRNA decay process is being referred to as target-directed miRNA degradation (TDMD) (24,25). In *C. elegans*, no trigger RNAs have yet been defined, and the rules of targeting a miRNA to TDMD may differ from that in other animals. Thus, EBAX-1-mediated decay of miR-35 and its sister depends exclusively on an intact seed sequence (nucleotides 2∼8 from the miRNA 5′ end), suggesting that a more limited duplex formation with a trigger RNA suffice for decay of at least this family of miRNAs.

**Figure 1.**
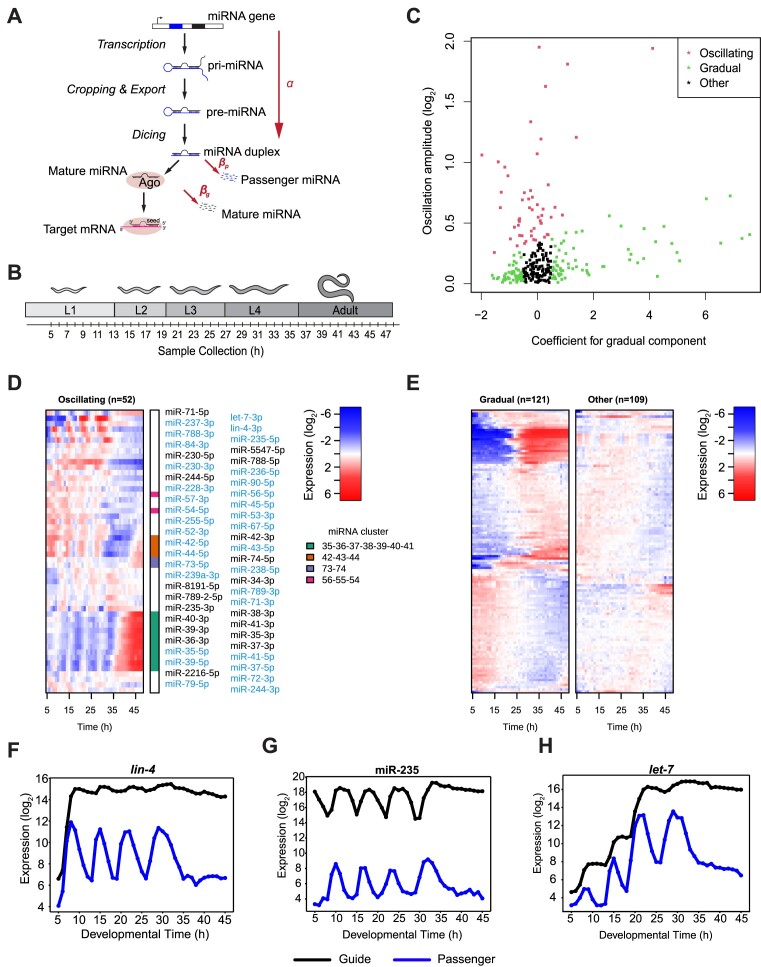
Temporal dynamics of miRNAs throughout larval development (**A**) Schematic depiction of miRNA production and decay. $\alpha$ and $\beta$ indicate the parameters used in the linear mathematical model to describe production (transcription and processing until miRNA duplex formation) and decay, respectively, as further described in the main text. (**B**) Schematic representation of the experimental design of the small-RNA time course. Cultures were sampled every hour from 5 to 48 h after plating synchronized L1 larvae on food, thus covering the course of larval development into early adulthood. (**C**) Scatter plot comparing the gradual coefficient (x-axis) and the amplitude (y-axis) from the cosine fit. Each dot represents a miRNA. The dots are colored according to three distinct groups of miRNAs: *oscillating* (red), *gradual* (green), and *other* (black). (**D**, **E**) Heatmaps for the three classes showing mean normalized and log_2_‐transformed expression levels of miRNAs over time. Each row corresponds to a specific miRNA, while each column represents a timepoint. The rows are sorted by 1D-tSNE in each panel separately. For the class of oscillating miRNAs, passenger strands are labelled in light blue, and the guide strands are labelled in black. The four miRNA clusters are color-coded in a separate column next to the heatmap. (**F–H**) Examples of individual traces of miRNAs whose passenger strands are all in the *oscillating* class but whose guide strands exhibit *oscillating* (miR-235; (G), *gradual* (*let-7*; (H)) or *other* (*lin-4*; (F)) behavior.

To investigate miRNA dynamics during *C. elegans* larval development, we performed small RNA sequencing on synchronized worm populations sampled hourly over 43 h, from the first larval (L1) to the young adult stage. By employing mathematical modeling, we sought to distinguish between miRNAs whose dynamics can be explained by regulated production alone versus those whose dynamics would require regulation of mature miRNA decay. Here, production encompasses all steps upstream of miRNA duplex formation, e.g. primary miRNA (pri-miRNA) transcription, pri-miRNA-to-pre-miRNA processing through Drosha/Pasha, and pre-miRNA processing by Dicer (Figure 1A). Our experimental data revealed an unanticipated complex pattern of *let-7* accumulation, with near stepwise increases during each larval stage, yet this was recapitulated by a dynamic production model. Indeed, we can explain these dynamics by a combination of rhythmic transcription and stage-specific regulation of *let-7* precursor processing by the RNA-binding protein LIN-28. In contrast, accumulation dynamics of several other miRNAs, including miR-235, miR-71, miR-788, could not be explained solely through modulation of biogenesis rates. Focusing on the rhythmically accumulating miR-235, we provide evidence that temporal modulation of its decay rate by EBAX-1 contributes to the generation of its dynamic patterns. Additional miRNAs are also destabilized by EBAX-1, albeit in different time windows, consistent with regulation of EBAX-1 through other mechanisms, possibly involving specific ‘trigger’ RNAs. Taken together, our study provides insights into how distinct combinations of regulated transcription, processing, and decay shape miRNA accumulation during development and offers a resource for dissecting the developmental functions and modes of regulation of miRNAs.

## Materials and methods

### C. elegans growth and maintenance

The *C. elegans* Bristol N2 isolate was used as the wild type. Transgenic strains were outcrossed at least twice to the wild-type strain. The following strains were employed in this study:

**Table:** 

Designation	Genotype	Source
HW1993	*xeSi312[eft-3p::luc::gfp::unc-54 3′UTR, unc-119(+)] IV*	This study
HW1939	*xeSi296[eft-3p::luc::gfp::unc-54 3′UTR] II*	(26)
HW2765	*mir-235(n4504) I; xeSi296[eft-3p::luc::gfp::unc-54 3′UTR, unc-119(+)] II*	*mir-235(n4504) I* from CGC, This study
HW2280	*xeSi408[mir-235p::pest-gfp-h2b::unc-54] II*	This study
HW2770	*xeSi480 [mir-71p::pest-gfp-h2b::unc-54] II*	This study
HW2643	*mir-235(xe181[seed_mutant]) I*	This study
HW2766	*mir-235(xe181[seed_mutant]) I; xeSi296[eft-3p::luc::gfp::unc-54 3′UTR, unc-119(+)] II*	This study
HW2706	*lin-28(xe192) I; xeSi312 [eft-3p::luc::gfp::unc-54 3′UTR, unc-119(+)] IV*	This study
HW2837	*let-7(xe488[tbb-1p]) X*	This study
HW3527	*ebax-1 (tm2321) IV*	(27)
HW3584	*xeSi296[eft-3p::luc::gfp::unc-54 3′UTR] II; ebax-1 (tm2321) IV*	This study
HW3564	*mir-235(xe372[eft-3p]) I*	This study
HW3629	*mir-235(xe372[eft-3p]) I; xeSi296[eft-3p::luc::gfp::unc-54 3′UTR] II*	This study

### Transgenic reporter strain generation

To create HW2280, we sought to amplify a ∼2.4 kb region upstream of the miR-235 hairpin (I: 6164693–6164715) from N2 genomic DNA using the Gateway BP Cleanse II Enzyme mix (Thermo Fisher Scientific; 11789020). The primer sequences used in the PCR reactions to clone it to pDONRP4P1 were as follows:

GB123:GGGGACAACTTTGTATAGAAAAGTTGgcttcaatttgtattgttcagac and GB124: GGGGACTGCTTTTTTGTACAAACTTGCtgatcctgatatcttcggacg. However, the resulting amplicon was reproducibly ∼1442 bp, as it lacked sequence between I: 6164371 and I: 6163476, annotated to contain tRNAs (T09B4.t1 and T09B4.t2). The promoter sequence was cloned into the destination vector pCFJ150 containing *pest-gfp-h2b* tag according to MultiSite Gateway Technology (Thermo Fisher Scientific). Transgenic worms were obtained by single-copy integration into the ttTi5605 locus on chromosome II as described previously (28). We assume that the missing region is misannotated in the Ce10 genome assembly because the cloned promoter shows activity in the same tissues previously reported (29) and because the resulting *gfp* mRNA dynamics recapitulate those of the endogenous pri-miRNA (Supplementary Figure S6A). The sequence of the amplicon (promoter) is as follows:

GCTTCAATTTGTATTGTTCAGACCACTCCCTTTTTTAAGACGTACAGCTACAAATTCAGATCTGAACACGAAAATCCGTCAGTCAACTTGGTATTCTTCTTTTTTCGCATTTCTCTTTTGCTTCTTCCAAAACGCATCCAGTTACATGTATCGTTTATGTGTTATTGGTATACTATGGCATTATGTACGCGCTTCACTTCTCCGTCACAAGGTGCTGGTGGACGGACGATGTTTAAAAAAATGAAGCGACATGCATGTACTGCGGCTGCTGCTTCTGCTGTGGATTGAGATTAAGAAAAGCTTAAAACCGGTTCCGATCCGCATGTCCTTGGCGCGCGGCAAACTCTTTTTTGCAGCTTCGTTTCAGCGTGAATCGCTTTGTAATAAAACAACCTGTTAGCTGGTCATTTTGGTCACTTAATCGTGTTCACTGGTTCATTGAGAAGAGAAATAGGAGATGAAAGGGAAAACATCGCTTCATTCATCGTTATGTCATTGTGAAAGGATACACAAAAATGGGCGGCGCCAAGAAGAGGGTACTTATTGCGTTTGACTTCCTTAAGAATAGTCCTATTCCAAGTTTGACTACTGATTTAATTTTATTCTTTCTAGTAAATTTCTTGTAATTGTTTGAAGCGAGTTTAGATTTTATGTTATGAAGATATTCCGTTTTATAAACTTTTAAAAGCTAATTTTGTTTTTCGTAAACGTAGGCGCTCCTAGATTAGTTTAAAGATCAGTTATTACGATAAAAAAGTTCACATGGCATTTGTAGTGACCACGTGACGTACGTAGGTCGCCTTTAAGGCAACCATGCTATTGTTTCGTGCCTACCTTCAAATAAAATGTGAATAATACTAGTTCAGAACAATTTTAGTCCTGTGTGTTTATTTTCTTCTATTTTGAAGCACGATTTATCTGTGAATGTTGAATTTATGTACCACGTCTGATTATCGATGCTCATTTCCATAGAAGTTTAAAATAAATTAGATACAGGAATGCACATTCTTCTGAGTTTTCGTTAATCGAATATCTCGATTAAAACCTGCAGCAGTATCTAATATTTTTAATTGAACAAGTATTTCTCATCATCCTAGAAATTCTTCAAATGTTCATCTTTCCAATAGAACATACAAATCACACGACGAACGAACAATTCTTCGTCTTCAGTTTTTCTCAAATCAAATGGTGTGTATGTAACCAAACCAGACACCTTTCTCCTTTTCTTAGACACCATACAAAATGGAGAGTACCCGGAGAGAAACAAATGGGCGGACCTTCAATTGGTACCAATCGAAACTCGTTTTCTTCTAAGTGTATGTATGTGTGTCCTTTCTTCAGATGACACCACCTGTGGATGGTTCTTAATTTTCTATCTATTCCTTATTCTTCCAAGTGCTATCTATTCGTTTGCTGAAAATCGTCCGAAGATATCAGGATCA.

To create the transcriptional reporter for *mir-71p* expressing *pest-gfp-h2b*, we amplified the approximately 2 kb upstream genomic region of miR-71 from genomic DNA using the following primers:

SN8: gcgtgtcaataatatcactcCGCTCGCTCAACTCTTCATT

and SN9: ccatggctaagtctagacatCCTCTGTGTGTGAGCTAGTTTC

Subsequently, we inserted this amplified region into NheI-digested pYPH0.14 using Gibson assembly (30). pYPH0.14 generated by *Not*I digestion of destination vector, pCFJ150 and addition of *Nhe*I::*pest-gfp-h2b* as described previously (26). The sequence of *mir-71* promoter is as follows:

CGCTCGCTCAACTCTTCATTCTATTTCTTCTACAGCAGTACTCTCCGTTTTTCAATGTTGTTTCGTCGTTTCATTCGGGTTTTTTGTTCACTTTTGTGAGTGGTGTGTTTCCGTGAGCGTGAGTGTGAGTGAGCTGTGCAGCGCCCTTGGACGACTACGACCGAAGGAAGGCGATATTGTGCTGATGCTGCCTGCTCGATCGTCGGTTTGACGACGAAGACAAGGACGACGATGCACACGAAACGAAATAGTGTGAAGCCGACGACGAGCGGACTAGCGCGCAGAAAATGGCATGTCTGATACCTCACGTGCCTCACGTAGTGTCTTCGTTGCCCTCGGGCCCCAATGGCGCTCGCTCACAGCGAGAGACGCACACACAAACATGTATACAAAAATGAAAGAAGAGAGAGAGACAGAGTATAACGAAAAATTGGCTGAGCGTGTGCCCCAGCAGGGCGCTCACGGCGCTCCGCTCAAGGCATATAACTCTGGTAATCTCTCCACACTCCTTTGTCACATTTATCTCTCGCTGCCACACTCACTTTTCTGGCGCTCCTCTCTTTGCTGGTACACGTGGCCAAATGCGCTCACCGAAAAGAGGGCGGAGCCGTGAGATGTCGTTGACGGGCGCCGCGTCTCGGTGTGAGGCTGGGCGCCGATGATTCTCCGCTGCTGCCTGCGTGTTTGGTTGTCTCTGCTCTCTCTGCTCCCTCTCTCGCCTCGCCCCGGGCATAGGGCTCCGCCTCTTTTAGTATGTTGTTGGCTGTTGAGGCCCTTTCTGTTGGCCTGTGTCGTCGCTGTGGTGCTGGTGTGTTGTGTTGTGTGTGCGTTTTTTGTGCGTGCGGGTGAGTGCGCGGCGCCTTTCCTCGCCGTTGAGCGTATTTTTGTGCCATACGGGAAAGAGGAAGGTGTCCTCCTGCACTTTACCCATCCTAAAAGCCTTCACCACAGCATCAACACTGAACTGTATTTTCTCCCTGAATATTCTTCTGTTACCTACTGTGATCTGGAATTCTAGAAAATTCAAGATCTTTCATCCTATGGCAGTACCTAAAAAGCTAGATATTAGTTGCCAGGCATGCATGAGGTAAGAAGCATCGTAAGGAGCTAGTTCAGCGTGTCCATAGAAACGAGTTGAAATAAGAAAATTTTAGTGAACGGAGATTCCGATACTCACCGTGTTCGTAGGCGTTCCCGGCATTTGTCCACCCACTAAGCTTCAGAAAATGAAAATTTAAATGCCTAAGAATGTATAGACCGCACTAATGTTCTACGTTTTCGCAGCAGAAAGAGGTGCCATTTGCAAGTTGAAGGTATTGCTCTATACCTTCTAAATTCCAACGCAAAGAGTCAATCAGGATTCTCCAAATCGTTTTTCTTTCTGAAGAAATACACGCCCCCTTTCCAAATCATCATTTTCGTATCCAAACAACGCCTAGAACCGAAGTTATAGCATCGACTTTTAGAGTGTCGTCGTTGCATTTTTTCTTTGCTGCGATGTTTGTATGTTTCTGTGCCTCCTTCCTCTTCTCTGCTCACTCCACCGATGCGCCCTCTTTCTCTCAGCCTTTGTGCTCTCTGATAGTCTCGTCGCTCTGCTTCTCCTCCCTGCCGCCGCGACCTCATTTTTTGCTTGCTTGCTCGCTTGCAAGGGTGGTGGGGTCGAAATTGAGAATGTGTTTTTTATGCGGAGAGATGAAGAGAGAGTGAGAAAGTGAGCGCGCGCGCGTGTTGAGCACTGGATGACGTTAGGTTAAGCTTCAGCCTTTTTTCCGAGCTGATAACACTAAAAGTTCATGTTATAGTGTGTATACTGATAAGGGGTAGAAGTAGAAGCTCAATAAAGATACAAACCATTCTTTGTCAATCATTCTGTCATATTCATCTACCGTATTCGCTGTACTTTCCAACAAAACCTCAGTGTTGAAACTAGCTCACACACAGAGG.

Transgenic animals for both *mir-235* and *mir-71* promoter fusion reporters were obtained by mos1- mediated single copy-insertion (MosCI) of the respective clones into the ttTi5605 locus (MosSCI site) on chromosome II (31). Both the strains were backcrossed thrice.

### Genome editing

Mutations in the endogenous *mir-235* locus were obtained by CRISPR-Cas9 to generate the *mir-235(xe181)* allele. For generation of sgRNA plasmids, we cloned the sgRNA sequences (mir-235sgRNA1 sense and antisense; mir-235sgRNA2 sense and antisense) into *Not*I-digested pIK198 plasmid (32). The sgRNA oligos and the HR repair template (miR-235 HR oligo 2) were obtained from IDT and are listed in Supplementary Table S2.

Wild-type worms were injected with a mix containing 50 ng/μl pIK155, 100 ng/μl of each pIK198 with a cloned sgRNA, 20 ng/ μl repair oligo for gene of interest (miR-235 HR oligo 2), co-CRISPR mix containing 100 ng/ml pIK208 and 20 ng/ml AF-ZF-827 (33) PAGE purified oligo (IDT). Single F1 roller progeny of injected wild-type worms were picked to individual plates and the F2 progeny screened for deletions using PCR assays with primers GB130, GB137 and GB138 (Supplementary Table S2). After DNA sequencing analysis to confirm the presence of the desired mutation, the alleles were outcrossed three times to the wild-type strain.

For generation of HW2837, the endogenous *let-7* promoter was replaced with the *tbb-1* promoter by CRISPR/Cas9-mediated editing using a previously published protocol (34). A 1 kb part of *let-7* promoter (1306–257 bp upstream of *pre-let-7*) was replaced by a 450 bp sequence upstream of the *tbb-1* start codon. The *tbb-1* promoter was amplified using two sets of primer pairs – a pair of short primers (JKc1 + JKc2) and a pair of primers carrying 120 bp overhangs complementary to the sequence flanking the integration site (JKc3 + JKc4) (Supplementary Table S2). The obtained PCR products were melted and reannealed to obtain an asymmetric DNA donor according to the following cycling conditions before adding it to the injection mixture: 95°C–2 min, 85°C–10 s, 75°C–10 s, 65°C–10 s, 55°C–1 min, 45°C–30 s, 35°C–10 s, 25°C–10 s, 4°C-forever and used as the homology-directed repair template. The endogenous *let-7* promoter was excised using two crRNAs (JKcr1 and JKcr2) and the F1 transformants were selected based on the expression of two co-injection plasmids, pRF4 *(rol-6 (su1006))* and pIK127 (*eft-3p::gfp::h2b::tbb-2 3′UTR)* (32). The injection mix was prepared freshly and was performed in N2 animals. First, 250 ng/μl of of Alt-R S.p. Cas9 Nuclease V3 (IDT, Cat # 1081058), 100 ng/μl of Alt-R® CRISPR-Cas9 tracrRNA (IDT, Cat # 1072532), 28 ng/μl each of JKcr1 and JKcr2 were incubated at 37°C for 10 min to form the RNP complex. Then a total of 4 μg of the hybridized asymmetric DNA donor and co-injection markers, piK127 (10 ng/μl) and pRF4(*rol-6(su1006)*) (40 ng/μl) were added to final volume of 20 μl filled up with water. 30 animals were injected and F1 transformants were screened and picked based on expression of GFP and roller phenotype. The F2 progenies were screened by PCR using JKg19, JKg23 and JKg5, JKg7 primer pairs (Supplementary Table S2) and outcrossed thrice.

To create HW2706, a 3.2 kb deletion encompassing the *lin-28* locus (deletion flanked by 5′-GTGCTACCG-3′ and 5′-ACCTACCTC-3′) was achieved by CRISPR/Cas9-mediated editing using the previously published *dpy-10(cn64)* co-conversion protocol (33). For generation of sgRNA plasmids, we cloned the sgRNA sequences (JKsg13 + 14) and (JKsg15 + 16) into *Not*I-digested pIK198 plasmid (32). The sgRNA oligos were obtained from IDT and are listed in Supplementary Table S2.

The injection was performed in wild-type animals which were injected with 10 ng/μl gBlock, 100 ng/μl sgRNA plasmids each, 20 ng/μl AF-ZF-827 (33), 50 ng/μl pIK155 and 100 ng/μl pIK208 (sgRNA targeting *dpy-10* in pIK198).

For generation of HW3564, the endogenous *mir-235* promoter was replaced by the *eft-3* promoter by CRISPR-Cas9 editing (35). The crRNAs (SN_cr1 and SN_cr2) designed to excise the *mir-235* promoter, were resuspended to the concentration of 0.4 μg/μl. The following *eft-3* promoter sequence:

GCACCTTTGGTCTTTTATTGTCAACTTCCATTGGTTCTTCCATTGTTTCTGTTAAATTAATGAATTTTTCATAAAATAAAGACATTATACAATATAAAAATGAAGAATTTATTGAAAATAAACTGCCAGAGAGAAAAAGTATGCAACACTCCCGCCGAGAGTGTTTGAAATGGTGTACGGTACATTTTCGTGCTAGGAGTTAGATGTGCAGGCAGCAACGAGAGGGGGAGAGATTTTTTTGGGCCTTGTGAAATTAACGTGAGTTTTCTGGTCATCTGACTAATCATGTTGGTTTTTTGTTGGTTTATTTTGTTTTTATCTTTGTTTTTATCCAGATTAGGAAATTTAAATTTTATGAATTTATAATGAGGTCAAACATTCAGTCCCAGCGTTTTTCCTGTTCTCACTGTTTAGTCGAATTTTTATTTTAGGCTTTCAACAAATGTTCTAACTGTCTTATTTGTGACCTCACTTTTTATATTTTTTTAATTTTTAAAAATATTAGAAGTTTCTAGGATAATTTTTTCGACTTTTATTCTCTCTACCGTCCGCACTCTTCTTACTTTTAAATTAAATTGTTTTTTTTTCAGTTGGGAAACA flanked by sequences of the *mir-235* endogenous genomic locus on the 5′ CAGTCAACTTGGT and 3′ CTTTGCTCATTG sides were synthesized as gBlocks® Gene Fragments (IDT) and was used as a template for homologous recombination (Supplementary Table S2). The gBlock was melted and re-annealed according to the following cycling conditions before adding it to the injection mixture: 95°C–2 min, 85°C–10 s, 75°C–10 s, 65°C–10 s, 55°C–1 min, 45°C–30 s, 35°C–10 s, 25°C–10 s, 4°C–forever. The injection was performed in N2 animals with the following injection mixture prepared freshly before the injection.

First, 250 ng/μl of of Alt-R S.p. Cas9 Nuclease V3 (IDT, Cat # 1081058), 100 ng/μl of Alt-R® CRISPR-Cas9 tracrRNA (IDT, Cat # 1072532), 28 ng/μl each of SN_cr1 and SN_cr2 were incubated at 37°C for 10 min to form the RNP complex. Then a total of 400 ng of the hybridized SN_gBlock2 (100 ng/μl) and co-injection markers, piK127 (10 ng/μl) and pRF4(*rol-6(su1006)*) (40 ng/μl) were added to final volume of 20 μl filled up with water. Thirty animals were injected and F1 transformants were screened and picked based on expression of GFP and appearance of roller phenotype. F2 progenies were genotyped by PCR using SN34, SN115 and SN116 primers (Supplementary Table S2) and outcrossed twice.

### Time course experiments

Several time course experiments (TC1-11) were performed in this study. The details for different time courses are given in Supplementary Table S3.

For TC1 to TC9, the following experimental method was used:

Gravid hermaphrodites of the respective worm strains were bleached to obtain eggs, which were then hatched in M9 buffer (in the absence of food) during an overnight incubation on a rotating wheel. The resulting arrested L1 larvae were transferred onto 2% Nematode Growth Medium (NGM) agar plates at a density of 2000 worms/plate with food (*E. coli*, OP50). The worms were grown at 25°C and were collected at an hourly interval from 18 to 30 h/31 h (for TC7); numbers of collected animals ranged from 2000 to 12,000 depending on the timepoints. After harvesting and three washes with M9 buffer, the worm pellet was resuspended in 1ml of Tri Reagent (Molecular Research Center; TR118) and snap-frozen in liquid nitrogen.

For TC10, synchronized L1 worms were grown on 2% NGM plates, seeded with *Escherichia coli* OP50 at 25°C. The worms were collected hourly from 12 until 34 h into M9 buffer. The worm pellet was resuspended in Tri Reagent (Molecular Research Center; TR118) and frozen in a 2 ml screw cap micro tube (Sarstedt; 72.693.005) containing ∼100 μl of 0.5 mm dia. Zirconia/Silica beads (BioSpec; 11079105z) in liquid nitrogen.

For TC11, synchronized L1’s of HW1993 (*xeSi312[eft-3p::luc::gfp::unc-54 3′UTR, unc-119(+)] IV*) and and HW2706 (*lin-28(xe192) I; xeSi312 [eft-3p::luc::gfp::unc-54 3′UTR, unc-119(+)] IV*) worms were grown in a liquid culture with E. coli OP50 bacteria (OD = 2.7 in S-Basal) at 25°C. Worms were collected hourly from 0 h until 40 h. A sample of worms was transferred from the liquid culture flask to a 15 ml conical tube, washed three times with M9 Buffer and finally the worm pellet was resuspended in Tri Reagent (Molecular Research Center; TR118) and frozen in liquid nitrogen.

### RNA isolation

For TC1-9 and TC11, the worms were lysed by five freeze/thaw cycles. For TC10, the worms were lysed at 4°C by bead beating in FastPrep-24™ grinder (SKU: 116004500) with 5 cycles of 8.0 m/s for 25 s followed by a pause of 120 s. Subsequently, worms were centrifuged at 16,000 g for 20 min at 4°C to remove debris and supernatant was transferred to a new tube. For TC1-TC3, TC5 and TC10, RNA was extracted and treated with DNAse I as described previously (36). For the other time courses (TC4, TC6-TC9, TC-11), RNA was isolated using Direct-zol™ RNA MicroPrep kit (Zymo Research; R2062) according to the manufacturer's protocol. On-column DNAse digestion was carried out for 15 min at room temperature. RNA was subsequently eluted in 15-30 μl of Ambion^TM^ nuclease free water (Thermo Fisher Scientific, AM9937).

### Small RNA sequencing

For different time courses, different sequencing libraries were prepared according to the manufacturer's instructions. The details of library preparation and sequencing instrument are listed in Supplementary Table S3. For TC11 (timepoints 0-23 h), three sequencing reactions were performed with different library preparations. First, sequencing libraries were generated using Illumina TruSeq Small RNA Library Prep Kit according to the manufacturer's instructions. The library was sequenced on Illumina HiSeq2500, as 51 cycles single-end reads. Since the reads obtained were not sufficient, the libraries were reprocessed using CleanTag Small RNA library preparation kit (Trilink Biotechnology) according to manufacturer's instructions. The library pool was size selected on a gel (140–160 bp) to enrich for the small RNA fraction. Subsequently, library was sequenced on NextSeq 500 as 76 cycle single-end reads, and then resequenced on NextSeq 500 as 51 cycle single-end reads to detect *let-7* passenger strand.

### Processing of the *C. elegans* small RNA-seq data

The following methodology was used to process data of TC1, TC6, TC7, TC9, TC11. The small RNA-seq fastq files were adapter trimmed (TGGAATTCTCGGGTGCCAAGG) at the 3′ end using the function preprocessReads() from the QuasR (37) package and mapped to the *C. elegans* genome (ce10) with the R package QuasR (37) using the alignment algorithm Bowtie (38). The command used to perform the alignments was ‘proj ←qAlign(’samples.txt‘,’BSgenome.Celegans.UCSC.ce10‘,maxHits = 100)’. For miRNA quantification, gene annotations from miRBase v22 were used and lifted over to ce10. We extended the genomic coordinates by ±3 bp (upstream and downstream) of the 5′ end of each miRNA and counted all the reads that started within those regions using the following command: ‘qCount(proj,matureMirsExt,orientation=’same’). To normalize for sequencing depth, each sample was divided by the total number of reads and multiplied by the average library size. Finally, the count table was log_2_-transformed after the addition of a pseudocount of 8 (log_2_(*x* + 8)) to minimize large changes in expression caused by low count numbers.

### Processing of the *C. briggsae* small RNA-seq data

The small RNA-seq data from *C. briggsae* (TC3) were processed the same way as the ones for *C. elegans*, using the *C. briggsae* genome assembly WS225 and miRNA gene annotation from MirgeneDB 2.0 (cb4) (39).

### RNA sequencing and processing

In TC7, for N2 and *ebax-1(tm2321)* mutants, mRNA was sequenced from samples collected from 18–30 h at 25°C as described above. Sequencing libraries were generated using Illumina TruSeq Stranded mRNA Library Prep Kit according to the manufacturer instructions. The RNA-seq samples were mapped to the *C. elegans* genome (ce10) with the R package QuasR (37) using the spliced alignment algorithm HISAT2 (40). The command used to perform the alignments was proj ←qAlign(‘samples.txt’,‘BSgenome.Celegans.UCSC.ce10’,splicedAlignment = TRUE,aligner=‘Rhisat2’). For gene quantification, gene annotation from WormBase was used (WS220). The command used to create the expression count table was qCount(proj,exons,orientation=‘opposite’, useRead=‘first’). To normalize for sequencing depth, each sample was divided by the total number of reads and multiplied by the average library size. Finally, the count table was log_2_ transformed after the addition of a pseudocount of 8 to minimize large changes in expression caused by low count numbers.

### Inference of pseudo-time for the *ebax-1(tm2321)* mutant and wild-type time course mRNA-seq data

During *C. elegans* larval development, >3700 genes oscillate at the mRNA level (26). For all those genes, we previously calculated their peak phases, and those peak phases cover the full spectrum between 0 and 360°. Based on that prior knowledge, we developed a simple yet effective method to visualize oscillations and timing in a given mRNA-seq time course experiment. The key idea is to define gene sets that show orthogonal expression over time (i.e. genes that differ by 90° in peak phase) and to quantify the expression of those genes in a new RNA-seq time course experiment. We first split the 3739 genes into 4 gene sets, gene set 1 containing genes with a peak phase of 0° ± 45° (effectively 315°–360° and 0°–45°) and gene sets 2, 3 and 4 containing genes with a peak phase of 90° ± 45°, 180° ± 45° and 270° ± 45°, respectively. Then we calculated the average gene expression levels for those 4 gene sets in the *ebax-1(tm2321)* and wild type mRNA-seq time course data (after mean normalizing each gene) and subtracted the two anti-phase pairs (*x* = geneset1-geneset3, *y* = geneset2-geneset4) to obtain two orthogonal timing readouts (*x*, *y*) for each RNA-seq sample. Visualized in a scatter plot, this resulted in robust circles for both wild type and *ebax-1(tm2321)* (Supplementary Figure S10A). We estimated the wrapped pseudo time of every data point (*x*, *y*) by calculating the respective polar coordinate angle. Finally, we unwrapped the angle values to obtain the final pseudo time for each sample.

### Differential expression analysis in the *ebax-1(tm2321)* versus wild type small RNA time course experiment

The pseudotime estimates from the mRNA-seq experiment allowed us to compare the developmental timing of the wild-type and the *ebax-1(tm2321)* time course experiments (TC7). It showed that *ebax-1(tm2321)* mutant animals had a 3-h delay but progressed through development at roughly the same speed as the wild type. We therefore selected the time points 18-28 h in the wild type and 21-31 h in the *ebax-1(tm2321* mutant for differential expression analysis (11 time points from each condition). We chose to use a linear model approach based on B-splines to determine statistically the differentially expressed genes. First, we calculated a cubic B-spline basis with 6 components that were evaluated at the eleven time points (from the wt perspective) using the R function ‘bs(c(18:28,28:28),6)’ from the splines package. Then we defined the design matrix containing a total of 14 predictors, namely an intercept term, the 6 spline components, a group variable distinguishing *ebax-1(tm2321)* from wild type, and 6 interaction terms between the group variable and all the spline components (model.matrix(∼bspline*Group)). Using the functions lmFit(), eBayes() and topTable() from the R package limma, we tested which miRNAs were significantly impacted by strain (by comparing the full linear model described above to one omitting the strain and interaction terms). We chose an adjusted p-value of 0.01 as our threshold for differential expression.

### RT-qPCR of miRNAs

10–20 ng of total RNA was reverse transcribed using TaqMan MicroRNA Reverse Transcription Kit (Applied Biosystems; 4366596) and the following TaqMan MicroRNA Assays:

**Table:** 

miRNA name (miRBase ID)	TaqMan microRNA assay ID
cel-miR-235-3p	001370
cel-miR-235-5p	464338_mat
cel-miR-71-5p	242106_mat
cel-miR-71-3p	242651_mat
cel-let-7-5p	000377
cel-let-7-3p	477630_mat
cel-miR-788	462309_mat
cel-miR-788*	465056_mat
cel-miR-247-3p	004491_mat
cel-miR-247-5p	244154_mat
cel-miR-1-3p	000385
sn2841 (reference gene)	001759

The reverse transcription reaction was incubated at 16°C for 30 min, 42°C for 30 min and 85°C for 5 min. The resulting 1.3 μl cDNA was used as template for qPCR to quantify miRNAs of interest. qPCR was performed on either on Step one Realtime PCR (Applied Biosystems) or Roche® LightCycler® 480 machine depending on the number of reactions. The reaction contained TaqMan 2x Universal PCR Master Mix, no Amp Erase (Applied Biosystems; 4324018) and the above-listed TaqMan MicroRNA Assays. The PCR conditions were initiated with 2 min at 50°C, 10 min incubation at 95°C, followed by 40 cycles of 95°C for 15 s and 60°C for 60 s.

Average Ct values were obtained from three technical replicates for miRNA and the reference, sn2841 RNA. Delta Ct (dCt) was calculated by subtracting Ct values of sn2841 from target miRNA Ct values. Normalized expression was plotted as dCt*-(1) against all timepoints. To depict the mean normalized miRNA levels, we subtracted the average dCt value (calculated across all timepoints) from each individual timepoint value and then multiplied the result by -1. The following formula was used: -ddCt = - (dCt – dCt_mean_). All the RT-qPCR plots were generated in R using the *ggplot2* package.

### RT-qPCR of mRNAs

800 ng of total RNA was reverse transcribed to cDNA using ImProm-II™ Reverse Transcription System (Promega; A3800) with random primers according to the manufacturer's protocol. cDNA was diluted 1:10 to detect *lin-42, pri-mir-235, pri-mir-71, SL1-pri-let-7* and 1:1000 or 1:2000 to detect *actin* from either pair of primers listed below. (For SL1-pri-let-7, the JKq17 and JKq18 primer pair was used.) RT-qPCRs were performed using either the SYBR Green PCR master mix (Applied Biosystems, 4309155) or the PowerUp™ SYBR™ Green Master Mix (Applied Biosystems; A25776) on either Step one Realtime PCR (Applied Biosystems) or Roche® LightCycler® 480 machine, depending on the number of samples. Data were normalized to *actin* expression levels.

**Table:** 

Transcript name	Primer > (fwd and rev)	Forward sequence	Reverse sequence
lin-42c	Lin-42c_L1 and Lin-42c-R1	TCTTGTTCACGTGACCTTC	GGCTCCGTCTGGCATAGTAA
gfp	SN10 and SN11	CTTGTTGAATTAGATGGTGATGTT	ACAAGTGTTGGCCATGGA
pri-mir-235	SN22 and SN23	GACACCATACAAAATGGAGAGTACC	TCATCTGAAGAAAGGACACACATAC
pri-mir-71	SN24 and SN25	TCCGAGCTGATAACACTAAAAGTTC	AGCGAATACGGTAGATGAATATGAC
SL1-pri-let-7	JKq1 and JKq3	GGTTTAATTACCCAAGTTTGAGGC	CGCAGCTTCGAAGAGTTCTG
act	Act-1fwd and Act-1rev	GTTGCCCAGAGGCTATGTTC	CAAGAGCGGTGATTTCCTTC
act	JKq17_act-1 F2 and JKq18_act-1 R2	GTCGGAAGACCACGTCATCAA	AGGGTAAGGATACCTCTCTTGGA

### Confocal imaging

To assess the spatial expression of *mir-235*, we utilized mixed-stage HW2280 worms (*xeSi408[mir-235p::pest-gfp-h2b::unc-54] II*) grown at 25°C. For confocal microscopy analysis, L4 hermaphrodites were selected and mounted on a 2% (w/v) agarose pad and immobilized using a drop of 10 mM levamisole (Fluca Analytical, 31742). Imaging of the worms was performed using an Axio Imager M2 upright microscope equipped with a Yokogawa CSU W1 Dual camera T2 spinning disk confocal scanning unit controlled by Visiview 3.1.0.3 software. Both DIC and fluorescent images were captured using a 40×/1.3 oil immersion objective, with exposure times of 10 and 300 ms, respectively. Subsequently, the acquired images were processed using ImageJ (Fiji) software after selecting representative regions.

### Time-lapse imaging

We used a transcriptional reporter of *mir-235* (HW2280: *xeSi408[mir-235p::pest-gfp-h2b::unc-54] II*) driving destabilized GFP to investigate the dynamic expression pattern with time-lapse imaging. The sample preparation for single worm imaging was done as previously described (26). Briefly, chambers were made of 4.5% agarose in S-Basal medium and OP50 bacteria was distributed onto the chambers by scraping it off from agar plates containing OP50. Single eggs were then placed on the chambers and flipped into glass coverslip surrounded by silicone insulator. Low melting agarose (3.5%) was used to seal the edges of the chamber and subsequently mounted on a glass slide for imaging. We imaged using 2× sCMOS camera model (T2) with an Axio Imager M2 (upright microsocope) equipped with a CSU_W1 Yokogawa spinning disk with a 20x air objective (NA = 0.8). The 488 nm laser was set to 70%, with 10 ms exposure and a binning of 2. The brightfield and fluorescent images were taken in parallel with 2 μm step size and 23 images per z-stack for a total duration of 60h with 10 min time intervals. The single worm imaging data was analysed as previously described using documented workflows (41). To account for worm size growth during development, the GFP intensities were normalized to area.

### Luciferase assay

Luciferase assays were performed as described before (26). In brief, we used HW1939, transgenic worms which express luciferase gene constitutively and ubiquitously. The transgene was crossed to *mir-235(n4504)* and *mir-235(seed_mut)* respectively. Gravid adults of each strain were bleached, and single eggs were placed in individual wells of a flat-bottom, 384 well-plate (Berthold Technologies, 32505). Each well contained 90 μl of S-Basal mixed with food (*E. coli* OP50 of O.D_600_= 0.9) and 100 μM Firefly D-Luciferin, substrate-Luciferin (p.j.k., #102111). The plates were sealed with Breathe Easier sealing membrane (Diversified Biotech, BERM-2000) and luminescence was measured using a luminometer (Berthold Technologies, Centro XS3 LB 960), every 10 min for 0.5 s for 72 h in 20°C incubator. Ingestion of luciferin during feeding results in an increase in bioluminescence with a precipitous drop in signal during molting due to absence of food intake of the worm. The duration of molts, larval stages and intermolts are thus annotated as described before (26) with the help of Luciferase Assay Analyzer tool, ‘PyLuc’ developed in-house by L. Morales Moya. Statistics were calculated using the Mann-Whitney U-test of the scipy.stats.mannwhitneyu package and from statannotations.

### Mathematical simulations

Equations were integrated using the explicit Euler forward method, i.e.


\begin{equation*}{\hat{x}}_{n + 1} = {\hat{x}}_n + {\mathrm{\Delta }}t\ f\left( {{{\hat{x}}}_n,{t}_n} \right),\end{equation*}

where ${t}_n$ and ${\hat{x}}_n$ are the discretized values for time and gene expression, respectively, $f( {{{\hat{x}}}_n,{t}_n} )$ is the differential equation, ${\mathrm{\Delta }}t$ is the time step. Note that the integration was always performed using the values in linear scale. Parameters were ${\beta }_g$, $\mu$ and ${\hat{x}}_0$.

To avoid numerical instabilities arising from the integration of real data, the time series for the passenger strand were linearly interpolated to increase the sampling rate 100 times. The solution was later down-sampled back to the original sampling rate.

To obtain the parameter set for which the solution the resembles the actual data, the error between the solution and the guide was minimized, i.e.


\begin{equation*}{E}_k = \ \mathop {\min }\limits_{{\beta }_g,\mu ,\ {{\hat{x}}}_0} \sum {\left( {{x}_n - {{\hat{x}}}_n} \right)}^2\end{equation*}

The minimization was performed in two steps: first, a grid search was performed over a plausible parameter range. These values were later refined using a minimization algorithm with such values as initial estimates, using the python function *minimize* from the package *scipy* (1.5.2). Appendix 1 contains a full derivation of the model and describes its testing against synthetic data.

## Results

### Dynamic changes in miRNA expression profiles during larval development

To investigate miRNAs dynamics occurring at short timescales during *C. elegans* larval development, we sampled a synchronously growing worm population frequently and over an extended period to sequence small RNAs. We plated synchronized populations of L1 larvae on food, grew them at 25°C, and collected samples hourly between 5 h and 48 h after plating, i.e. all the way to adulthood (Figure 1B). We mapped the reads to miRNAs using annotations from miRBase v.22 (42) and designated them as ‘5p’ and ‘3p’, respectively, depending on the strand of the pre-miRNA from which they derived. For 130 pre-miRNAs, we were able to detect expression of both 5p and 3p strands, whereas for the remaining 22 pre-miRNAs, we detected only one strand. In the following, we will use the term guide strand (or miR) to refer to the more abundant or exclusively detected strand; and ‘passenger’ strand (or miR*) to refer to the less abundant strand (based on mean expression levels over the whole time-course).

During the same period of *C. elegans* development, a substantial part of mRNA dynamics can be captured by two broad patterns, oscillating and gradually changing (26,36). Visual inspection of miRNA expression revealed similar patterns (Supplementary Figure S1A), prompting us to emulate the previous mRNA-seq analysis by fitting miRNA expression patterns using a linear model (36). For the fit, we only used timepoints within the oscillatory window (8–35 h after plating). The model contained two oscillatory components (cos and sin) with a fixed period of 7 h, and a linear component to capture gradual changes in the expression over time. We thus obtained the amplitude, the peak phase, and the gradual coefficient for each miRNA. The gradual component was used to capture the miRNAs that increased or decreased steadily during development. We then plotted the amplitude versus the coefficient for the gradual component to identify *oscillating* (red dots) and *gradual* (green) miRNAs (Figure 1C). Based on empirically chosen cut-offs, out of 282 miRNAs detected (including both passenger and guide strands), we assigned 52 to the *oscillating* class (18.4%) and 121 (42.9%) to the *gradual* class (Figure 1D, E); this latter class included miRNAs with both increasing or decreasing levels over time. Among the *gradual* miRNA, we specifically noticed a sizeable group of miRNAs that showed an increase in expression in a particular time window, ∼25 h after plating (Supplementary Figure S1B,C). Among the *oscillating* miRNAs, 33/52 (63%) were passenger strands (labeled in blue in Figure 1D), although these constituted only 130/282 (46%) of all detected miRNAs. As explained in more detail below, this enrichment seems consistent with the fact that passenger strands are less stable than the guide strands (17). Finally, 109 miRNAs (38.6%) belonged to neither class; these were classified as *other* (Figure 1C, black dots). We note that as we analyze RNA from whole animals, we may fail to observe dynamics that are specific to a subset of cells in which a given miRNA is expressed, so that we may underestimate the overall extent of dynamics. Moreover, these data cannot distinguish concentration changes occurring synchronously across all cells that express a given miRNAs from those that result from an increase or decrease in the number of cells that express a certain miRNA.

We observed similar dynamics for miRNAs transcribed from physically adjacent miRNA genes, so-called clusters (Figure 1D, Supplementary Figure S1). For instance, among the eight members of *miR35fam*, expression patterns are most similar among the seven clustered members, miR-35∼41, whereas the pattern of the eighth member, miR-42, is more like that of its co-clustered miRNAs, miR-43 and miR-44. Notably, all eight *miR-35fam* members exhibited rhythmically changing levels in larvae, and generally accumulated to substantial levels despite accelerated decay of the abundant embryonic *miR-35fam* pool during transition to the L1 stage (21). These observations suggest that, in addition to their well-known function in embryos (43,44), *miR-35fam* miRNAs may have a post-embryonic function.

We also noticed a striking deviation from the general principle of shared dynamics among clustered miRNAs. Specifically, although miR-359 and miR-785-3p are separated by <400 nt and thought to be co-transcribed (29), miR-785-3p is expressed at largely invariant levels throughout the time course, whereas miR-359 is only present at relatively low levels during the first ∼25 h and then, around the time of the third molt, increases steeply (Supplementary Figure S2). Although not further investigated by us, this suggests that the two miRNAs are not co-transcribed and/or that miR-359 is subject to specific post-transcriptional regulation.

We examined the patterns of guide strand accumulation for miRNAs whose passenger strands we had classified as *oscillating*. This revealed strikingly different behaviors for different miRNAs. For *lin-4*, levels of passenger and guide strand were low at the first sampled time point, 5 h after plating, consistent with transcriptional silencing prior to this point (29,45–48), but then increased rapidly (Figure 1F). However, whereas lin-4* exhibited high-amplitude oscillations, the relative amplitude was much reduced, i.e. oscillations damped, for the much more abundant *lin-4* guide strand whose levels remained quite stable after reaching a first peak by 9 h. This difference appears intuitively consistent with differences in stability of the two species, where increased stability of the guide strand will at the same time increase average levels and decrease amplitude (Supplementary Figure S3A,B). Accordingly, we were surprised that for miR-235, both strands exhibited similar relative amplitudes of oscillations, although guide strand levels were three orders of magnitude above that of the passenger strand (Figure 1G). Equally surprisingly, the two strands exhibited large phase-differences in their accumulation patterns. We revisit the issue of how these miR-235 dynamics are generated below.

Finally, whereas the let-7* levels oscillated, the *let-7* guide displayed a near stepwise increment in accumulation until reaching a plateau by ∼30 h, i.e. at the early L4 stage (Figure 1H). Based on detection by Northern blotting, accumulation of *let-7* had previously been reported to initiate at the L3 stage (4,8,9,46,49). However, consistent with rhythmic *let-7* transcription during each larval stage (7,49), the greater sensitivity and dynamic range of small RNA sequencing that we employ here reveals that mature *let-7* begins to accumulate already during the first larval stage. Taken together, we find that for oscillating passenger strands, their matching guide strands can exhibit diverse behaviors. Since the biogenesis of guide and passenger strand from a given locus is intertwined, i.e. transcription, pri-miRNA and pre-miRNA processing are shared production events, these findings suggest an important contribution of events downstream of production in shaping miRNA fates.

### The dynamics of miRNAs can be captured by a mathematical model invoking time-varying production rates

To investigate the origin of the miRNA dynamics that we had observed, we employed mathematical modeling, utilizing ordinary differential equations (ODEs). The observed abundance of miRNAs is generally determined by their production and decay rates, where the latter is defined as the combination of both degradation and dilution rates. Hence, a simple model can be formulated to elucidate the change in miRNA levels, as described by the following equation, i.e.


(1) \begin{equation*}\frac{{dX}}{{dt}} = \alpha \left( t \right) - \beta X,\ \end{equation*}

where $dX/dt$ is the rate of change in miRNA, $X$ is the concentration of the miRNA, $\alpha ( t )$ is the production rate and $\beta$ is the decay rate. We note that this model is agnostic as to whether changes happen at the cellular or organismal level, e.g. whether we observe increased miRNA abundance over time because all cells that previously expressed a miRNA now express more of it, or because more cells start to express it.

Since both passenger ($p$) and guide ($g$) come from the same transcript, the model assumes the same production rate, $\alpha ( t )$, for both strands. Note that ‘production’ in this context encompasses transcription as well as the subsequent processing steps up to the point of the guide:passenger duplex formation (Figure 1A). However, differential dynamics could emerge from different decay rates ${\beta }_p$ and ${\beta }_g$, respectively. Biologically, this may for instance be imposed through protection of the guide strand from nucleases by binding to AGO (Figure 1A).

Hence, the equation becomes as follows:


(2) \begin{equation*}\left\{ {\begin{array}{@{}*{1}{c}@{}} {\frac{{dp}}{{dt}} = \alpha \left( t \right) - {\beta }_pp}\\ {\frac{{dg}}{{dt}} = \alpha \left( t \right) - {\beta }_gg} \end{array},\ } \right.\end{equation*}

where ${\beta }_n$ are the respective decay rates. If the degradation rate for the passenger strand is sufficiently large with respect to the frequency, the passenger levels can adapt rapidly enough to the changes due to the oscillations to approximate the production rate as $\alpha ( t ) = \ {\beta }_pp$ (see Appendix), and thus both equations can be combined into a single one, i.e.


(3) \begin{equation*}\frac{{dg\left( t \right)}}{{dt}} = {\beta }_p \cdot p{\mathrm{\ }}\left( t \right) - {\beta }_g \cdot g\left( t \right)\end{equation*}

However, measured passenger and guide levels might depart from real values due to technical biases. We assumed these biases to be proportional to the levels of each strand, causing differential amplification based on the number of molecules and thus preferentially affecting the more abundant strand. To reflect this assumption, we multiplied passenger levels by a constant $k$ in Eq. (3). As $k$ and ${\beta }_p$ are both constants, they were combined into a single parameter $\mu$, which we named scaling factor. Thus, the final equation is given by


(4) \begin{equation*}\frac{{dg\left( t \right)}}{{dt}} = \mu \cdot p\ \left( t \right) - {\beta }_g \cdot g\left( t \right)\end{equation*}

We modelled the levels of the guide strand by integrating numerically Equation (4) for a set of parameter values using the forward Euler method. The optimal set of parameters, $\mu$ and ${\beta }_g$, were obtained by minimizing the difference between estimated and real values of the guide strand. We explored the limitations of this approach using synthetic data, which showed that it performs well for passenger strand half-life at or below ∼5 min and at least 5 times lower than the guide strand half-life (Appendix), a requirement likely met in most cases (19). We note that the model explicitly assumes that decay rates are constant over time. As such, and as explored further below, an inability to obtain a good model fit for a given miRNA may hint at regulated decay.

### A combination of rhythmic transcription and regulated processing generates a near-stepwise accumulation pattern of *let-7*

**Figure 2.**
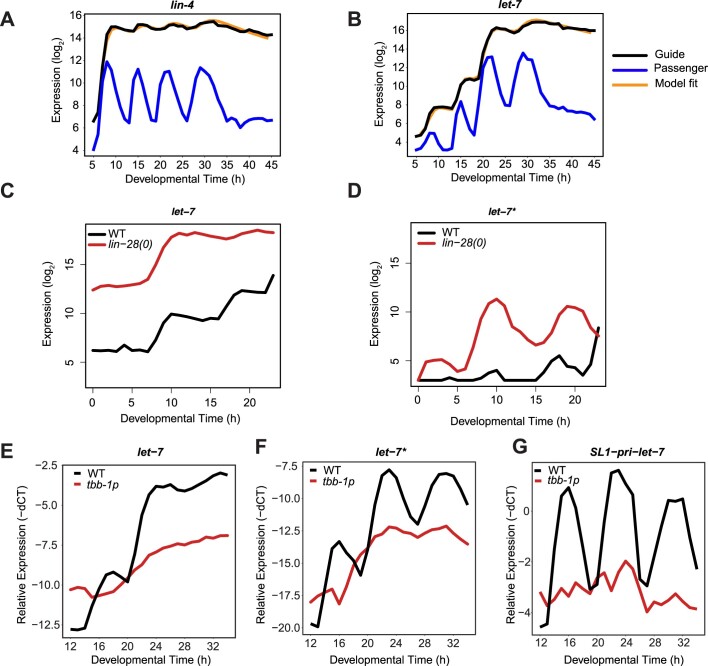
Mathematical modelling to predict dynamics of guide miRNAs. (A, B) The dynamics of *lin-4* (**A**) and *let-7* (**B**) can be explained by the linear model (Eq. 4). *Black*: guide strand, *blue*: passenger strand and *orange*: best fit for guide. (C, D) The dynamics of *let-7* (**C**) and let-7* (**D**) in wild-type (WT) and *lin-28(0)* animals quantified by small-RNA sequencing. Synchronized L1 larvae were placed in liquid culture with food at 25°C and sampled at the indicated times. Animals developed more slowly in this than in the separate experiment in (A, B), involving growth on plates. (**E–G**) The dynamics of *let-7* (E) and let-7* (F) and *SL1-pri-let-7* (the trans-spliced primary miRNA) in WT and *tbb-1p::let-7* worms quantified by RT-qPCR. Synchronized L1 larvae were plated on food at 25°C and sampled hourly at the indicated times after plating roughly covering end of L1 through mid L4. Reactions of RT-qPCR were performed in technical triplicates, the values were averaged and normalized to the reference gene. TaqMan assays were employed for assessing the small nucleolar RNAs sn2841 as a normalization factor, while β-actin served as the reference gene in the case of SL-1-pri-let-7. The –dCT values were calculated as follows: –dCT = –(miRNA/mRNA CT values – reference CT values).

We applied the model to the sequencing data that we had obtained (Supplementary Table S1, Supplementary Figure S3C). We note that the model is agnostic as to whether changes in abundance occur across all or only a subset of cells. For instance, an increase in abundance of a miRNA over time may reflect an increased expression in all cells of a larvae, or the initiation of production in cells that did not previously increase it. Strikingly, the model effectively captured the very distinct behaviors of *lin-4* and *let-7* (Figure 2A and B). Although not ruling out additional regulatory mechanisms, this result suggested that that their unique dynamic patterns could be explained through differences in their production rates. Indeed, although *let-7* had a longer inferred half-life than *lin-4* (8.7 h versus 6.3 h), swapping their decay rates did not qualitatively alter their respective patterns of accumulation (Supplementary Figure S4A, B).

We hypothesized that the unique behavior of *let-7* may instead result from a combination of rhythmic transcription and post-transcriptional control of pri-miRNA or pre-miRNA processing. As the RNA-binding protein LIN-28 was previously shown to regulate these processing events for *let-7* (49,50), we tested the effect of loss of LIN-28 on *let-7* dynamics. Consistent with a role of LIN-28 in repressing mature miRNA accumulation upstream of strand separation, we observed elevated levels of both *let-7* guide strand and *let-7* passenger strand (denoted let-7* in the following) (Figure 2C and D). Moreover, although we continued to observe rhythmic peaking of let-7* strand levels at ∼9 and ∼18 h, *let-7* guide strand levels reached a precocious plateau by ∼9 h, with no subsequent step at the time of the next let-7* peak. In other words, loss of LIN-28 leads to an accumulation of *let-7* specifically in the L1 stage, when LIN-28 is also most abundant (51) (We note that expression starts at elevated baseline relative to wild-type animals. We attribute this to *let-7* promoter activity in embryos (29) and a role of LIN-28 in preventing mature *let-7* accumulation even in embryos.)

In a complementary experiment, we replaced the endogenous *let-7* promoter with a constitutive *tbb-1* promoter. In this scenario, we no longer observed pri-*let-7* or let-7* oscillations (Figure 2F and G). Regulation by LIN-28 appeared to remain intact, as both *let-7* and let-7* levels increased during the second larval stage after 16 h (Figure 2F and G), when LIN-28 is downregulated (51). Furthermore, our approach found that despite the different dynamics between conditions, the inferred guide degradation rate was the same (Supplementary Figure S4C, D). Collectively, these data support a model where a combination of rhythmic transcription and regulated processing through LIN-28 generate the striking pattern of *let-7* accumulation.

### Modelling predicts additional regulation beyond production for guide miRNAs

**Figure 3.**
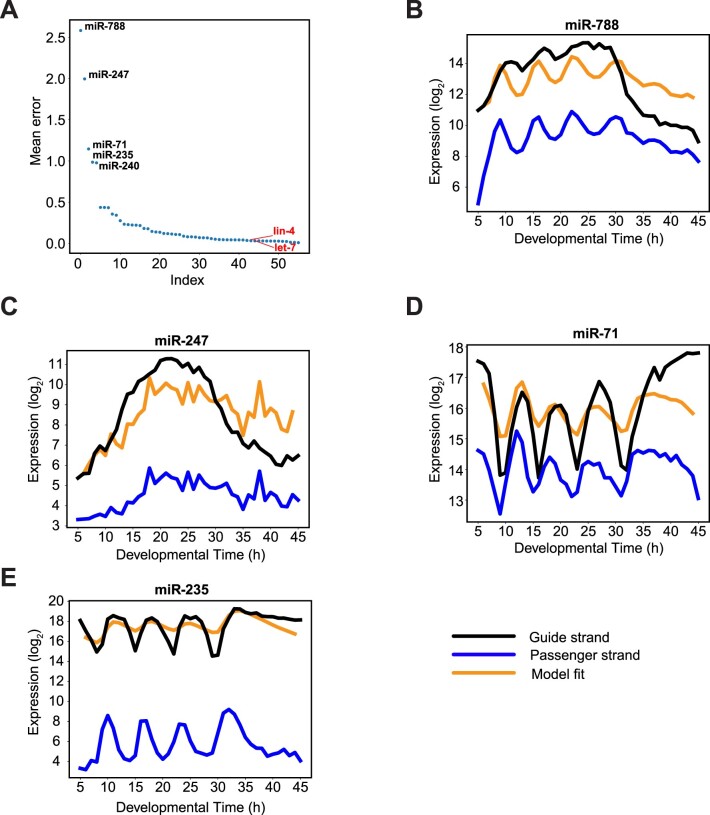
Regulation of guide miRNA accumulation after the dicing step predicted by mathematical modelling. (**A**) Least squared error for each miRNA whose expression was modelled. miRNAs exhibiting the five largest errors (i.e. poorest model fit) as well as *lin-4* and *let-7* are labeled. (**B–E**) Experimental and modelled dynamics for the indicated miRNAs with poor model fit: *Black*: guide strand, *blue*: passenger strand and *orange*: best fit for guide.

Time-variable decay rates are thus not a necessity to generate complex patterns of miRNA dynamics, and we observed a good model fit for many miRNAs (Figure 3A, Supplementary Table S1). However, we identified several examples where a model with time-invariant decay rates was unable to accurately predict the dynamics of miRNAs. The expression patterns of these miRNAs were validated by RT-qPCR in independent time-courses, excluding detection biases (Supplementary Figure S5). Specifically, miR-788 and miR-247 displayed rapid declines after 30 h, not explained by the initial model (Figure 3B and C). For miR-71, the model failed to explain the observed combination of larger relative amplitudes and higher levels of the guide relative to the passenger strand (Figure 3D). miR-235 exhibited repeated rapid declines in levels, resulting in a large relative amplitude, and the shape of its expression pattern deviated greatly from that of the passenger strand. This combination of features, which we examine in more detail below, could not be predicted by the model (Figure 3E). Hence, these miRNAs appeared subject to additional regulatory steps, downstream of Dicer.

### miR-235 is transcribed from a rhythmically active promoter

**Figure 4.**
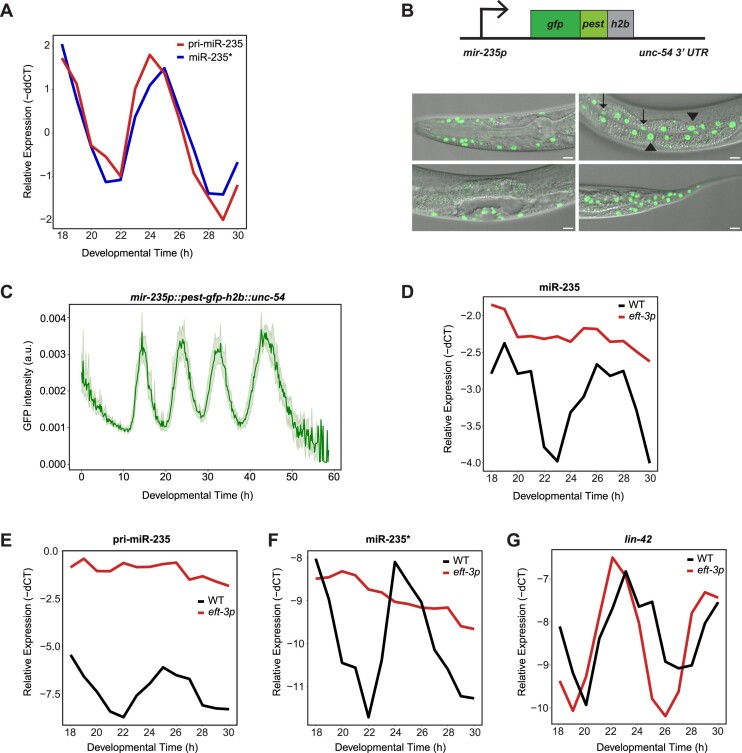
Rhythmic *mir-235* promoter activity promotes rhythmic miR-235 guide strand accumulation. (**A**) Quantification of endogenous pri-miR-235 and miR-235* levels by RT-qPCR. Synchronized L1 wild-type larvae were plated on food at 25°C and sampled at the indicated times after plating. (**B**) *Top:* Schematic of *mir-235* transcriptional reporter. *Bottom:* Confocal micrographs of *mir-235p::gfp::pest::h2b::unc-54* L4 larvae reveals expression of *mir-235* in head, seam, hypodermis, vulva, Pn.p cells and tail. Strain HW2280 (xeSi408[*mir-235p::pest-gfp-h2b::unc-54] II*) was generated by MosSCI. (**C**) Quantification of GFP levels of the reporter by time-lapse live imaging throughout larval development, starting from hatch (left panel). Green line indicates average GFP intensities with ± 95% confidence interval (light green shading, *n* = 23). (**D–G**) RT-qPCR quantification of the indicated RNA species in animals carrying an endogenous substitution of the *mir-235* promoter by a constitutive *eft-3* promoter (*mir-235(xe372[eft-3p]) I*). Synchronized L1 larvae were plated on food at 25°C and sampled at the indicated times after plating.

Given its particularly unusual dynamics, we sought to explore the mechanisms that contribute to rhythmic accumulation of miR-235 in more detail. Oscillating mRNA levels are driven by rhythmic transcription (41) and, using RT-qPCR for quantification, we confirmed that pri-miR-235 and miR-235* exhibited rhythmic accumulation with similar relative amplitudes and phases (Figure 4A). To examine *mir-235* promoter activity more directly, we fused a ∼1.4 kb of genomic region upstream of pre-miR-235 to DNA encoding destabilized, nuclear-localized green fluorescent protein (*pest-gfp-h2b*) and the *unc-54_3′UTR_*, considered devoid of regulatory sequences (Figure 4B). This type of reporter, integrated into chromosome II by Mos1-mediated single copy integration (31), allows detection of dynamic promoter activity (41). The promoter was active in skin, specifically in seam cells, hypodermis, Pn.p cells and vulva, as well as in unidentified head neurons and the tail (Figure 4B). With this reporter, we could assess how accurately the transgene's promoter activity reflected the levels of endogenous primary and passenger miRNA. Thus, we plated synchronized larvae on food at 25°C and sampled hourly from 18–30 h. We observed that the *gfp* mRNA recapitulates the primary miRNA levels with highly similar periods, amplitudes, and peak phases (Supplementary Figure S6A), implying that oscillations in pri-miRNA levels are caused by rhythmic promoter activity. Moreover, the dynamics of the miR-235* (passenger strands) recapitulated well the observed dynamics of their corresponding pri-miRNAs and *gfp* mRNA, corroborating the use of passenger strands as proxies for transcription (pri-miR-235 levels) in our models. We also confirmed for a second rhythmically accumulating miRNA, miR-71, that passenger strand dynamics effectively recapitulated those of the cognate pri-miRNA, or *gfp* mRNA transcribed from the putative *mir-71* promoter (Supplementary Figure S6B).

We next used time-lapse imaging to examine the temporal *mir-235* promoter activity in individual worms that we tracked over the entire period of larval development. We observed rhythmic GFP accumulation, with four oscillatory peaks corresponding to the four larval stages (Figure 4C).

To determine the functional relevance of rhythmic *mir-235* promoter activity for rhythmic miRNA accumulation, we replaced the *mir-235* promoter, in the endogenous locus, with a constitutive *eft-3* promoter. We confirmed by RT-qPCR that this manipulation abrogated rhythmic transcription of pri-miR-235 as both the pri-miRNA and the miR-235* passenger strand accumulated constitutively and, relative to wild-type animals, at elevated levels in the *eft-3p::mir-235* strain (Figure 4E, F). The amplitude of miR-235 guide strand accumulation was greatly decreased (Figure 4D). This was not a result of a general loss of developmental and/or gene expression synchrony as confirmed by direct measurements of developmental progression (Supplementary Figure S6C) and the observation of sustained rhythmic accumulation of a control mRNA, *lin-42*, in the *eft-3p::mir-235* strain (Figure 4G). We conclude that the rhythmic transcription from the miR-235 promoter is necessary for rhythmic miR-235 guide strand accumulation.

### Oscillatory expression of miR-235 is conserved in *C. briggsae*

**Figure 5.**
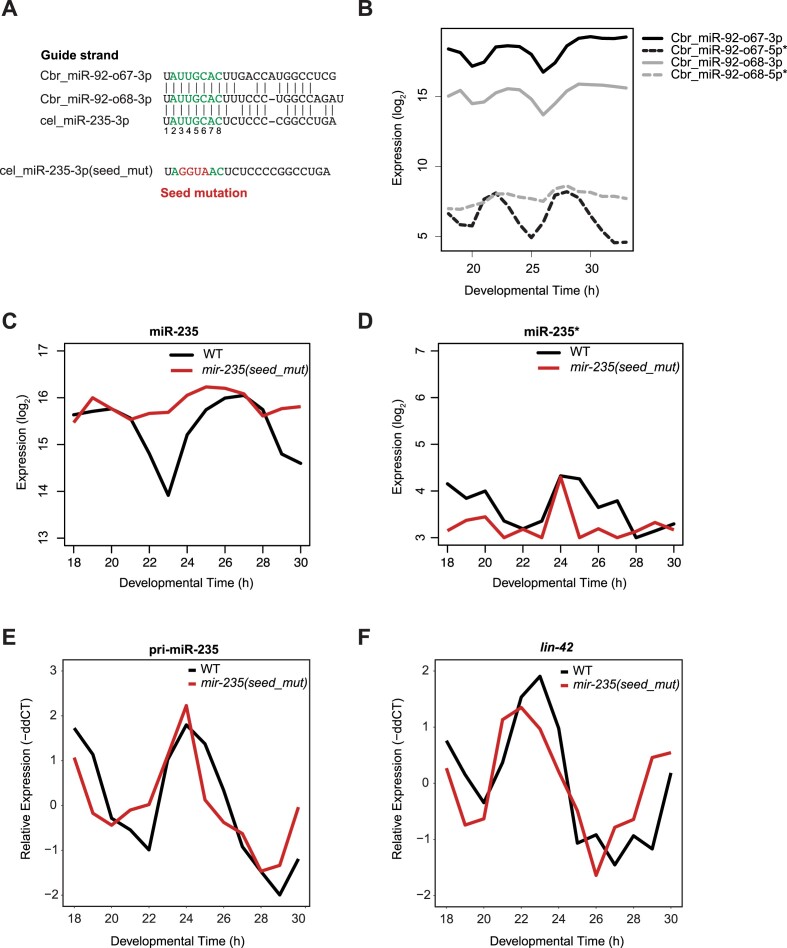
The seed sequence of miR-235 promotes oscillations (**A**) Sequence alignment of miR-235 in *C. elegans* and its orthologues in *C. briggsae*. The seed region is shown in green (nt 2–8). Seed mutation introduced into cel-miR-235–3p are shown in red. (**B**) log_2_ transformed expression of miR-235 paralogs in *C. briggsae*. Synchronized L1 larvae were plated on food at 25°C and sampled at the indicated times after plating to sequence small RNAs. (**C**, **D**) log_2_ transformed expression of miR-235 (**C**) and miR-235* (**D**) in N2 (black) and *mir-235(seed_mut)* (red) quantified by small-RNA sequencing. (**E**, **F**) Mean-normalized expression of pri-miR-235 (E) and *lin-42* (F) quantified by RT-qPCR. Reactions of RT-qPCR were performed in technical triplicates, the values were averaged and normalized to the reference gene. TaqMan assays for the small nucleolar RNAs sn2841 was used as the normalization control for miR-235 and miR-235*. The reference gene used for pri-miR-235 and *lin-42* was β-actin. The following equation was used to quantitate the relative expression: –ddCT = –(dCT – dCT_mean_). dCT was calculated as follows: dCT = (miRNA CT values – reference CT values)

Although rhythmic *mir-235* promoter activity appears necessary for high-amplitude rhythmic accumulation of miR-235, the mathematical model and the observations of *lin-4* and *let-7* (which also undergo rhythmic transcription but exhibit different guide strand dynamics from miR-235 (Figure 1D)), indicate that it is not sufficient to explain the dynamics. Thus, to investigate features further contributing to the unusual miR-235 dynamics, we exploited evolutionary conservation. In *C. elegans*, miR-235 is the sole orthologue of the mammalian miR-92 family, whereas in *C. briggsae*, two paralogous miRNAs, termed Cbr_miR-92-o67 (Cbr_miR-235b) and Cbr_miR-92-o68 (Cbr_miR-235a) exist (Figure 5A). Although Cbr_miR-92-o68 and Cel_miR-235 share an identical sequence across nearly the entire guide strand, with only three nucleotides difference, Cbr_miR-92-o67 is more dissimilar from either, particularly outside the seed region. To determine whether either or both of the *C. briggsae* miRNAs also accumulated rhythmically, we performed a short time course experiment covering *C. briggsae* development hourly between 18 and 33 h of larval development at 25°C. Strikingly, we observed similar rhythmic expression profile for both paralogs of the miR-235 guide (Cbr_miR-92-o67_3p and Cbr_miR-92-o68_3p). As in *C. elegans*, the Cbr_miR-92-o67_5p passenger strand oscillated nearly in antiphase to its guide strand, and the Cbr_miR-92-o68_5p passenger strand lacked any obvious rhythmic accumulation pattern altogether (Figure 5B). We take these findings to imply a potentially conserved mechanism of miRNA regulation post-production that may depend on the extended seed sequence (nt 1–9) and/or seed-distal region (nt 16–19), shared among the three miRNAs.

### Oscillations and stability of miR-235 are seed-dependent

To test our inference that the seed of miR-235 contributed to its dynamic accumulation, we mutated four nucleotides in the seed region of endogenous *C. elegans* miR-235 by genome editing (Figure 5A). We also introduced complementary mutations in the miR-235* to maintain the hairpin structure of the precursor for processing.

Like *mir-235(n4504)* deletion mutants, the *mir-235(xe181[seed_mutant])* animals appeared overtly wild-type with only minor variations in developmental tempo (Supplementary Figure S7). Hence, we could grow synchronous cultures of wild-type and *mir-235(seed_mut)* animals to examine miR-235 dynamics by small-RNA sequencing. A time course that covered 18 h through 30 h after plating revealed a striking change in the dynamics of the mutant miRNA: oscillations were damped, and it appeared that specifically the steep decline beginning at ∼21 h in the wild-type strain failed to occur in the mutant strain (Figure 5C). By contrast, both miR-235* and pri-miR-235 continued to accumulate rhythmically, with large amplitudes, in the mutant animals (Figure 5D, E, Supplementary Figure S8). Thus, these findings support a role of the seed sequence in generating miR-235 dynamics and specifically suggest that they increase miRNA decay during certain periods of development.

### EBAX-1 regulates miR-235 dynamics in larval development

**Figure 6.**
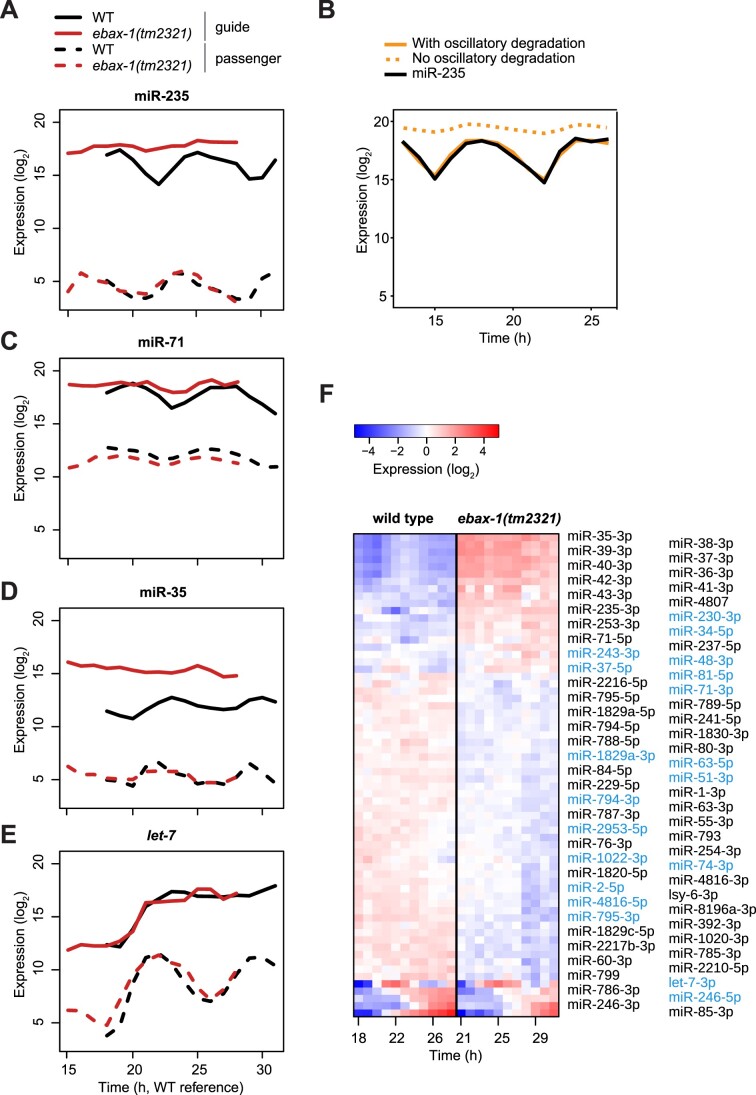
EBAX-1 dependent miRNA changes (**A**) Expression of *mir-235* in WT and *ebax-1(tm2321)* samples determined by small-RNA sequencing. Synchronized L1 larvae were plated on food at 25°C and sampled hourly between 18 and 30 h after plating. The x-axis represents pseudo time obtained from the RNA-seq experiment of the same samples (see Materials and methods). The y-axis represents log_2_ expression levels. (**B**) Mathematical modelling of miR-235 levels between 13 and 26 h in WT using a non-linear function (see Appendix). Measured data are indicated in solid black, the predicted levels in solid orange. The effect of EBAX-1 loss was modelled (dashed orange) by setting of the oscillatory degradation to zero, retaining only basal degradation. (**C**–**E**) Expression of the indicated miRNAs as in (A). (**F**) Heatmap of miRNAs differentially expressed between WT and *ebax-1(tm2321)* (see Methods). Each row corresponds to a miRNA, with passenger strands labelled in *light blue* and guide labelled in *black*. The columns represent the time points of 18–28 h in WT and 21–31 h in *ebax-1(tm2321)*, which roughly match in developmental time (see Supplementary Figure S10B)

Given this tentative evidence for regulated miR-235 degradation, we wondered whether EBAX-1 was involved in this process. To test this, we synchronized populations of wild-type and *ebax-1(tm2321)* mutant animals by hatching embryos in the absence of food and sampled every hour from 18 h through 30 h after plating them on food. Extracted RNA was then subjected to small RNA sequencing. Since we had found that wild-type and *ebax-1(tm2321)* mutant animals differed in developmental tempo (Supplementary Figure S9), we also performed mRNA expression profiling to determine the developmental pseudo time of each sample *in silico* (see Methods, Supplementary Figure S10A). This analysis revealed a 3 h delay in the *ebax-1(tm2321)* mutants relative to the wild type (Supplementary Figure S10B). We thus shifted the *ebax-1(tm2321)* experimental time by -3 h for correct alignment in time. Consistent with our hypothesis of EBAX-1-mediated degradation, we noticed a remarkable accumulation of the miR-235 guide (cel-miR-235-3p), accompanied by a reduction in oscillation amplitude, in the mutant relative to wild-type animals (Figure 6A). Specifically, we observed a consistently high level of miR-235 in the mutant animals at times when its concentration would decrease in wild-type animals, suggesting rhythmic episodes of degradation. Consistent with this notion, we found that we could qualitatively recapitulate the wild-type miR-235 accumulation dynamics in a model that combined rhythmic production with rhythmic degradation (Figure 6B, Appendix). Moreover, elimination of rhythmic degradation from this model correctly predicted elevated guide strand levels that exhibited greatly damped oscillations (Figure 6B).

We found that the rhythmically accumulating miR-71 also displayed sensitivity to EBAX-1 regulation (Figure 6C), consistent with our inability to model its dynamics when assuming constant degradation (Figure 3A). Similar to the situation of miR-235, miR-71 guide strand levels were elevated, and its accumulation amplitude greatly reduced in *ebax-1(0)* mutant animals. Additionally, we observed increased levels of miR-35 family miRNAs upon EBAX-1 loss, revealing that this regulatory interaction extends beyond embryo-to-larva transition (21). Although EBAX-1-mediated miRNA decay appears thus relatively widespread in larvae, not all dynamically produced miRNAs were sensitive to EBAX-1 loss. For instance, *let-7* guide levels remained largely unaffected by loss of EBAX-1 (Figure 6E), consistent with a predominance of regulation at the level of production.

To assess the effects of EBAX-1 loss globally, we performed differential expression analysis (see Methods) considering only the developmental time interval that was covered by both N2 and *ebax-1(tm2321)* mutant (Supplementary Figure S10B). In addition to the upregulation observed for miRNAs whose degradation EBAX-1 mediates, this revealed a set of miRNAs whose levels decreased in *ebax-1(tm2321)* mutant relative to wild-type animals (Figure 6F). Although we have not explored this further, we suspect that this reflects indirect effects, resulting possibly from a change in the pool of available AGO proteins due to inappropriate accumulation of the EBAX-1-sensitive miRNAs, or from dysregulation of the targets of certain EBAX-1-sensitive miRNAs.

### EBAX-1 promotes decay of miR-788 and miR-247 in late larval development

**Figure 7.**
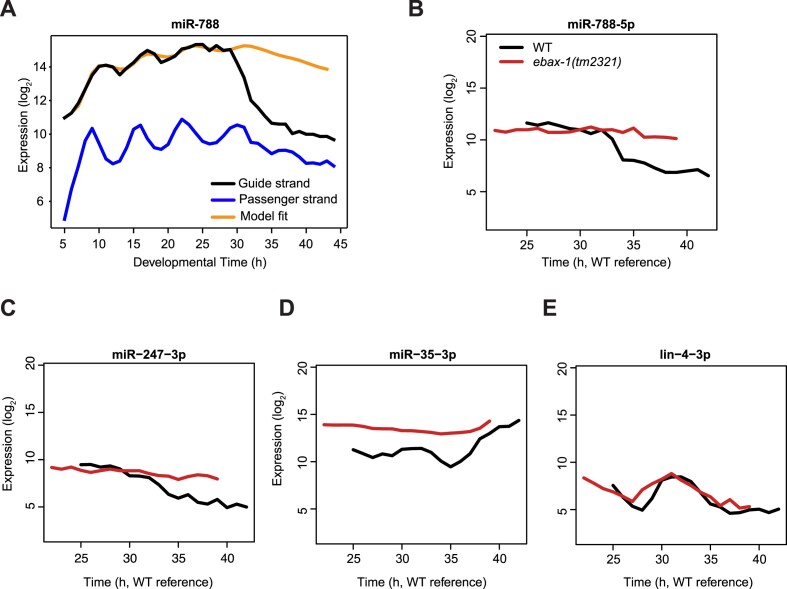
EBAX-1 dependent miRNA changes (**A**) miR-788 dynamics over time and the predicted data fitting up to 30 h. *Black*: guide strand, *blue*: passenger strand and *orange*: best fit for guide (from 5 h to 30 h). (**B–E**) Expression of miRNAs from small-RNA sequencing in WT and *ebax-1(tm2321)* samples. After being plated on food at 25°C, synchronized L1 larvae were sampled every hour from 25 to 42 h. Pseudo time from the RNA-seq experiment on the same samples is represented by the x-axis. The log_2_ expression levels are plotted on the y-axis.

Finally, we were intrigued by the fact that miR-788 and miR-247 showed rapid declines in their levels during the L4 stage, ∼30 h after plating on food (Figure 3B, C, Supplementary Figure S5D,E). Indeed, although we failed to recapitulate their dynamics using the simple model with time-invariant decay rates when we modelled their behavior over the entire time course, the model fit much better when we sought to fit dynamics only up to 30 h (Figure 7A). To examine whether the striking decrease in their levels beyond that point were caused by EBAX-1 activity, we sampled and sequenced another time course covering this late episode of development, ranging from 25 to 42 h after plating (late L3 through young adulthood). Similarly to the first sequencing time course, we again observed a ∼3 h-delay as revealed by simultaneous staging of animals at different timepoints and scoring for vulva morphology (Supplementary Figure S11A). Thus, we shifted the *ebax-1(tm2321)* experimental time by 3 h for correct alignment in time. We observed a striking stabilization of both miRNAs (Figure 7B, C), but not the passenger strand in the *ebax-1(tm2321)* mutant (Supplementary Figure S11B). Additionally, the experiment reproduced the effects of EBAX-1 loss on the oscillatory accumulation of miR-235 and miR-71, now at a later developmental stage again and without affecting the passenger strands (Supplementary Figure S11C and D). Finally, it appears that the levels of *miR-35fam* miRNAs in wild-type animals begin to approach those observed in *ebax-1(tm2321)* mutant animals upon transition to adulthood (occurring at ∼36 h after plating wild-type animals), suggesting that EBAX-1-mediated degradation of these miRNAs is reduced or altogether ceases at this time (Figure7D). Other dynamically expressed miRNAs, such as the rhythmically expressed passenger strand of *lin-4* (Figure 7E) and *let-7* (Supplementary Figure S11E), showed negligible change upon loss of EBAX-1.

Taken together, EBAX-1 is required for rhythmic episodes of miR-235 and miR-71 declines, decay of miR-788 and miR-247 during the L4 stage, and destabilization of *miR-35fam* throughout much or all of larval development. The fact that each of these miRNA reveals different temporal trajectories and responses to EBAX-1 loss is consistent with the notion that specific TDMD triggers, yet to be identified, control EBAX-1 activity in a miRNA-specific manner.

## Discussion

### Many miRNAs exhibit dynamic accumulation patterns during *C. elegans* larval development

Previous perturbation studies in L1-stage larvae and adult *C. elegans* revealed for most miRNAs long half-lives that exceeded the duration of a larval stage (16–18). Such long half-lives would be expected to curtail miRNA dynamics, delaying both downregulation and, upon increased production, settling into a new, higher steady-state level. Nonetheless, our data of miRNA expression during postembryonic development at unprecedented temporal resolution reveal numerous miRNAs with highly dynamic patterns of accumulation. We propose that several mechanisms help to reconcile these observations. First, as larval development is coupled to exponential biomass growth (52), miRNA decay during this period will be a combination of enzymatic degradation and dilution. The perturbations used to measure miRNA half-lives in previous studies are likely to disrupt body growth along with development, potentially reducing or eliminating the dilution component. Second, the applied perturbations may trigger secondary, compensatory effects, e.g. a general transcriptional block may limit the production of miRNA decay factors. Third, and particularly intriguing, miRNA decay rates may exhibit variations during development, providing a mechanism to effect more rapid changes in miRNA expression programs. This is a scenario that we demonstrate here for a subset of miRNAs.

### Exploring the dynamics of miRNAs through mathematical modelling

Temporally highly resolved sequencing data enabled us to formulate simple ODE based linear models to understand the source of distinctive patterns of miRNA expression. We used the expression levels of passenger strands as a proxy for production and predicted the levels of guide miRNAs. Strikingly, this simple model was sufficient to accurately capture the dynamics of many miRNAs, even for cases involving complex mechanisms of regulation, and thus accumulation patterns, as *let-7*, where we found that a combination of rhythmic transcription and early-stage post-transcriptional regulation by LIN-28 results in a near-stepwise pattern of accumulation across larval stages.

Although the ability to capture the observed behavior by the simple model does not rule out more complex modes of regulation, failure to do so implies the existence of additional modes of regulation not considered in the model. Hence, such failures were of particular interest to us. The most notable examples in our study were miR-247, miR-788, miR-71 and miR-235, all of which we show here to undergo periods of controlled degradation.

### Dynamic miRNA degradation in *C. elegans*

TDMD acts as a two-component system of miRNA decay, involving a trigger RNA and a general decay factor, represented by EBAX-1 (or its orthologues ZSWIM8 in mammals or Dora in flies). The trigger RNA thus plays a key role in identifying the decay substrate. Moreover, through its own expression dynamics, it can additionally constrain the domain of turnover activity in space and time (53,54). Yet, in most cases of ZSWIM8-, and all cases of EBAX-1-mediated decay, the triggers are unknown, and temporal regulation has remained unexplored. Thus, even in the well-studied case of *miR-35fam* decay at the embryo-to-larva transition (21), current data cannot distinguish between a constitutive role of EBAX-1 in destabilizing *miR-35fam*, which would render it more susceptible to transcriptional modulation, and a specific pulse of decay. Our data suggest that EBAX-1 destabilizes *miR-35fam* miRNAs constitutively throughout larval development, but the effect appears lessened or absent in adults.

Contrasting with *miR-35fam*, the four other miRNAs that we show to be regulated by EBAX-1, namely miR-235, miR-71, miR-788 and miR-247, all exhibit little if any constitutive elevation in *ebax-1* mutant larvae, and instead appear stabilized during a specific window of time, when their levels would normally decrease. Thus, the temporal pattern of EBAX-1 sensitive miRNAs differs among individual miRNAs. The question arises: How does EBAX-1 bring about distinct miRNA regulation? As *ebax-1* mRNA levels change rhythmically (26,36) a hypothetic rhythmic accumulation of EBAX-1 might contribute to the patterns of miR-235 and miR-71. However, these miRNAs reach troughs at different times, which is not easily reconciled with this model. Hence, we propose that activation of EBAX-1, possibly through rhythmic trigger RNA accumulation, could be the key to achieving dynamic degradation of these miRNAs. Similarly, the expression of distinct trigger RNAs during the L4 stage may activate EBAX-1 for degradation of miR-788 and miR-247.

### The features of TDMD triggers in *C. elegans*

In mammals as well as flies, TDMD/ZSWIM8-mediated miRNA decay requires extensive complementarity between the miRNA and the trigger RNA, such that both the miRNA seed and its 3′ end are duplexed (22,23,55,56). By contrast, mutagenesis of the EBAX-1 target miR-35 showed that only its seed sequence, but not its 3′ end sequence is required for EBAX-1-dependent decay and thus, presumably, trigger RNA binding (21).

Our data support that EBAX-1-mediated miRNA decay involves the seed sequence since mutation of the miR-235 seed prevented the miRNA’s rhythmic decrease (without substantially altering miR* and pri-miRNA dynamics). We have not explored whether sequences outside the seed are essential but note that nucleotides 16–19 are identical among all rhythmically accumulating miR-235 homologues in *C. elegans* and *C. briggsae*. Although it thus remains to be seen whether this sequence could contribute to decay, we highlight that even the two *C. briggsae* paralogues differ in their three 3′-terminal nucleotides, whose pairing to a TDMD trigger is crucial for efficient TDMD in mammals (25). Hence, it appears that the features of nematode TDMD triggers may indeed more generally deviate from those of their mammalian counterparts. Limited complementarity (relative to the situation in flies and mammals) may also provide an additional obstacle to computationally predicting potential TDMD triggers, possibly explaining why no such triggers have yet been identified in *C. elegans*. Indeed, and as noted previously (21), evidence for EBAX-1-mediated decay as a TDMD-mechanism is purely by analogy to the mammalian and fly system, and it remains possible that triggers in addition to or even distinct from RNAs are required to induce miRNA decay in worms.

### TDMD may control miR-92 family expression and rhythmic accumulation across animals

Considering the apparent differences in TDMD between *C. elegans* and other animals, it is striking that oscillatory accumulation of miR-235 is not limited to *C. elegans* and *C. briggsae*. This dynamic pattern extends to the orthologous miR-92a in *Drosophila*, which accumulates in specific neurons with a circadian, ∼24 h period to modulate neuronal excitability by suppressing SIRT2 in a rhythmic manner (57). While it is not currently understood whether rhythmic degradation contributes to this behavior, it is worth noting that miR-92a was identified as a putative Dora/ZSWIM8 target in *Drosophila* embryos (55). Moreover, miR-92a and miR-92b were found to be regulated by ZSWIM8 in mouse neurons, contact-inhibited murine embryonic fibroblasts, and mouse embryos (23,55,56). Hence, it will be of interest to understand whether miR-92 also accumulates rhythmically in specific mammalian cells, and whether the apparent similarities in expression dynamics and regulatory mechanisms reflect functional conservation or convergence.

### Temporally resolved miRNA expression data as a resource to understanding miRNA function

Beyond providing insight into the mechanisms that generate dynamic miRNA accumulation, and specifically the roles of miRNA decay, the extensive miRNA profiling dataset presented in this study may provide cues to the physiological functions of miRNAs. Thus, the rapid increase of *lin-4* during the L1 stage is understood to be the mechanism that drives transition from the first to the second larval stage by initiating repression of the *lin-14* mRNA (3,5). Our data reveal numerous other miRNAs whose levels increase dramatically during specific time windows, notably during the late L3 stage, around 25 h after plating synchronized L1 stage larvae. Examples include miR-357∼miR-358; miR-240∼miR-786; miR-359 and miR-85. This identifies a possible window of activity of these miRNAs. Intriguingly, previous promoter analysis (29) suggested expression of all of these miRNAs in spermatheca and/or uterus, among other cells, although not all miRNAs expressed in these tissues accumulate dynamically (including miR-785, which is in a cluster with miR-359 but exhibits constitutively high levels during development) (Supplementary Figure S2). Considering that the time of upregulation coincides with the occurrence of major morphogenetic events in the uterus and the initiation of spermatogenesis, our data thus implicate these miRNAs in these processes. We point out that the exact same samples for which we performed here small RNA sequencing were previously subjected to mRNA sequencing [TC2 in (26)]. The availability of temporally highly resolved, paired miRNA and mRNA data offers a powerful resource to explore these and other possible developmental functions, and mechanisms of regulation, of miRNAs in future studies.

## Supplementary Material

gkae115_Supplemental_files

## Data Availability

All RNA-seq data are accessible at NCBI’s Gene Expression Omnibus (58) under SuperSeries accession number GSE245904. The submission includes tables with raw and normalized expression values.
