# Supplementary material for: Dynamics of miRNA accumulation during C. elegans larval development
Source: Nucleic Acids Res. 2024 Feb 21;52(9):5336–55. doi: 10.1093/nar/gkae115 (PMC11109986; doi:10.1093/nar/gkae115)
Supplement: gkae115_Supplemental_files [file gkae115_supplemental_files.zip › Appendix_final.docx]

**Appendix**

The proposed computational approach, also known as the quasi-steady state [QSS] approach, was tested on its performance concerning the following factors: 1) How unstable does the passenger strand have to be to provide a good proxy for the input function (i.e., what is the minimal acceptable degradation rate). 2.) By how much does the degradation rate of the passenger strand have to exceed that of the guide strand to permit correct identification of the parameter values. 3) How well defined are the error landscapes for different values of parameters. Additionally, we performed an alternative modelling approach on *lin-4* to see how well the assumptions hold on the real data. Finally, we explored a model including time-variant degradation rates.

*Test on synthetic datasets*

The QSS approach was tested using a synthetic signal with a non-integer period similar to experimental measurements and sampled at intervals of one hour from 0 h to 44 h, i.e.

$\alpha\left( t \right)=2+\cos\left( \frac{2\pi}{6.8}t \right), t\in\left[ 0, 44 \right] (S1)$

First, we tested the quasi-steady state assumption for the estimation of the input function. For sufficiently large $\beta_{p}$, the input can be approximated as $\alpha\left( t \right)\approx\beta_{p}p$. To test this assumption, the passenger strand model was solved analytically, i.e.

$\frac{dp(t)}{dt}=\alpha\left( t \right)-\beta_{p}p\left( t \right), (S2)$

and the difference between the input and the passenger dynamics for a cycle was computed as

$E\left( \beta_{p} \right)=\frac{1}{A\cdot T}\int_{T} \left( \alpha\left( t \right)-\beta_{p}p\left( t \right) \right)^{2}dt, (S3)$

where $T$ and $A$ are the input period and maximum amplitude, respectively. For the input signal shown in equation S1, the error is given by
$E\left( \beta_{p} \right)=\frac{1}{1+\left( \frac{\beta_{p}}{\omega} \right)^{2}}=\frac{1}{1+\left( \frac{6.8}{2\pi}\beta_{p} \right)^{2}} (S4)$

We found that the assumption of $\alpha\left( t \right)\approx\beta_{p}p$ generally holds for half-lives under 12 minutes (Appendix Figure 1A), and the difference between the input function and the scaled passenger strand levels decreased to below 1% for values of passenger degradation rates of 10 h^-1^ or higher (corresponding to a half-life of ≤ 4 min).

Secondly, we tested by how much the passenger strand degradation rate has to exceed that of the guide stand to permit correct identification of the parameter values. To study the relationship between parameters, the passenger degradation rate was redefined in terms of the guide degradation rate such that $\beta_{p}=\gamma\beta_{g}$, i.e.

$\left\{ \begin{aligned} \frac{dp}{dt}=\alpha\left( t \right)-\gamma\beta_{g}p \\ \frac{dg}{dt}=\alpha\left( t \right) - \beta_{g}g \end{aligned} , \left( S5 \right) \right.$

Under the quasi-steady state approach, in which $\alpha\left( t \right)\approx\beta_{p}p$, the model simplifies as

$\frac{dg\left( t \right)}{dt}=\beta_{g}\left( \gamma\cdot p \left( t \right)-g\left( t \right) \right) \left( S6 \right)$

We compared the guide degradation rates obtained with the QSS approach for a synthetic dataset with a known value for $\beta_{g}$, for different values of the proportionality parameter $\gamma$. We found that the QSS approach can retrieve the correct value for the guide degradation rate for relatively small values of $\gamma$; i.e., even when the passenger was only five times more unstable than the passenger, we obtained accurate results (Appendix Figure 1B).

Finally, we explored parameter certainty: shallow valleys indicate a large parameter uncertainty while deep wells express a large confidence. While both the proportionality and the initial condition showed a similar shape for many parameter sets, the error function for the guide degradation rate changed qualitatively for different values. For the sake of simplicity, we chose a large value of proportionality ($\gamma=100$) and varied $\beta_{g}$.

For a large range of values of guide strand decay rates (between 0.05 and 5 h^-1^, corresponding to a range of half-lives from 13.9 hours to 8.31 minutes), minima can be recovered with a reasonable degree of confidence. However, for low (high) degradation rates the error function becomes shallow towards lower (higher) values, preventing a precise determination of the parameter value but still enabling an estimate of the highest (lowest) value.


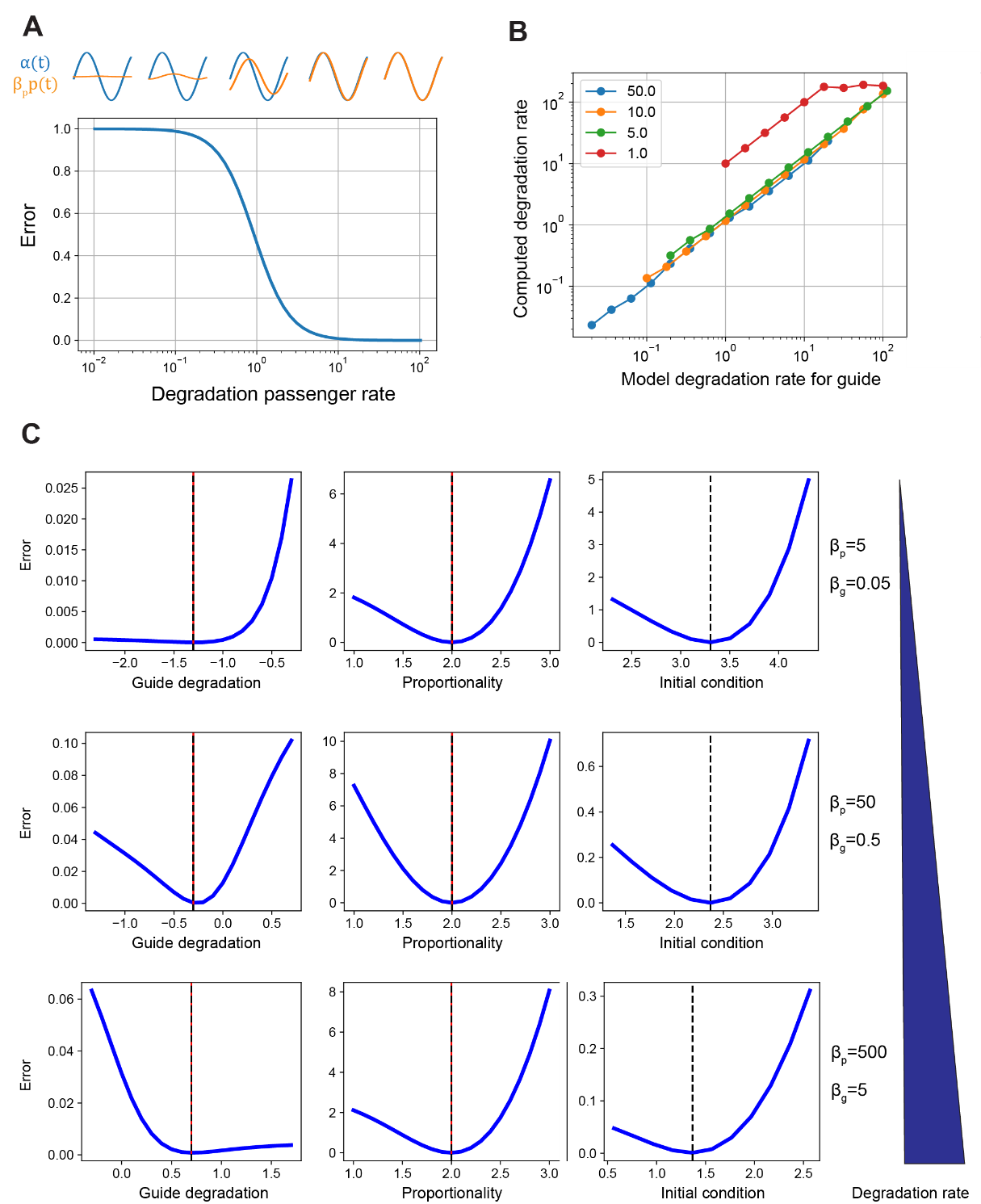


**Appendix Figure 1. Testing the QSS approach using synthetic data.**

**A)** Error (Equation S4) between the actual input function ($\alpha$) and the estimated input function from the scaled passenger strand ($\beta_{p}p$) for different values of the passenger degradation rate. Examples above the graph show the shape of the input function (blue) and the scaled passenger strand (orange) for different values of passenger degradation rate (0.01, 0.1, 1, 10, 100).

**B)** Estimated value for the guide strand using the QSS approach, using equation S6, compared to the real value of the model for different values of proportionality, shown as different colours.

**C)** Slices of the error landscape along different values of each parameter (different columns) while keeping the others fixed. Each row displays an increasing value of both degradation rates with a proportionality of $\gamma=100$. Solid red lines represent real values and dashed black lines represent estimated values. The triangle indicates increasing degradation rates from top to bottom.

*Test on real data*

To further test the validity of the quasi-steady state (QSS) assumption for the passenger strand, we pursued an alternative approach based on the estimation of the input function. To account for technical biases, the original model (Equation 2) was redefined to be

$\left\{ \begin{aligned} \frac{dp}{dt}= \alpha\left( t \right)-\beta_{g}p \\ \frac{dg}{dt}=k\alpha\left( t \right)-\beta_{g}g \end{aligned} , (S7) \right.$

where $\alpha_{p}$ and $\alpha_{g}$ are the input functions for both the passenger and the guide strands, respectively. The form for the biased differential equation can be derived if we assume that the measured value of the guide strand is some scaled value of the true value, i.e. $g=k\hat{g}$,

$\frac{dg}{dt}=k\frac{d\hat{g}}{dt}=k\left[ \alpha\left( t \right)-\beta_{g}\hat{g} \right]=k\left[ \alpha\left( t \right)-\beta_{g}\frac{g}{k} \right]=k\alpha\left( t \right)-\beta_{g}g (S8)$

Assuming that both strands come from the same precursor and that there are no additional layers of regulation, we would expect that $\alpha_{p}=\alpha_{g}$.

These input functions can be estimated based on the measured values of both strands and their derivatives, for a chosen value of parameters, i.e.

$\left\{ \begin{aligned} \alpha_{p}\left( t \right)=\frac{dp}{dt}+\beta_{p}p \\ \alpha_{g}\left( t \right)=\frac{1}{k}\left( \frac{dg}{dt}+\beta_{g}g \right) \end{aligned} , (S9) \right.$

Therefore, for a set of parameters, the difference between input functions can be used to define the following error

$E=\frac{1}{L}\int_{L} \left( \alpha_{p}\left( t \right)-\alpha_{g}\left( t \right) \right)^{2}dt, (S10)$

which can be rearranged as

$E=\frac{1}{L}\int_{L} \left( \frac{dp}{dt}+\beta_{p}p-\frac{1}{k}\left( \frac{dg}{dt}+\beta_{g}g \right) \right)^{2}dt, (S11)$

To find the optimal set of parameters, the error was minimised. To estimate the value of the derivative, the signal was differentiated using a first-order central differences method, i.e.

$\left. \frac{dx}{dt} \right|_{n}=\frac{x_{n+1}-x_{n-1}}{2\Delta n}, (S12)$

where $n$ is the chosen timepoint and $\Delta n$ is the sampling period between timepoints.

To illustrate the approach without the QSS assumption, we applied it to *lin-4*. We found a well-defined minimum for key parameters (Appendix Figure 2A,B). Yet, the relationship between the passenger degradation rate and the technical bias showed a large uncertainty due to a large correlation between parameters (Appendix Figure 2C). This supports the use of the alternative QSS approach, where both the technical bias and the passenger degradation rate are combined in a single parameter, as in Eq. 4.

The computed input functions for both strands matched to a reasonable degree of similarity (Appendix Figure 2D). Moreover, the computed input function for the passenger strand (scaled accordingly) and the experimentally observed levels for the passenger strand were highly similar (Appendix Figure 2E), showing that the assumption of $\alpha\left( t \right)\approx\beta_{p}p$ holds, thereby supporting the use of the QSS approach. Indeed, both models yield very similar estimates of µ (full model $\mu=\beta_{p}k=4.82 \left( h^{-1} \right)$, QSS approach $\mu=4.85 \left( h^{-1} \right)$, Appendix Figure 2G). Finally, a comparison of the outputs of the two models showed no significant difference for the predicted guide dynamics (Appendix Figure 2F for the full model and 2H for the QSS assumption), and a well-defined minimum was found for both the guide degradation rate and the scaling factor (Appendix Figure 2G). We conclude that the QSS is a reasonable assumption. Indeed, given that the numerical differentiation necessary for the full model introduces noise (illustrated by the observed distortions of the computed input functions in Appendix Figure 2D), we considered the QSS approach more suited to the present analyses.

**
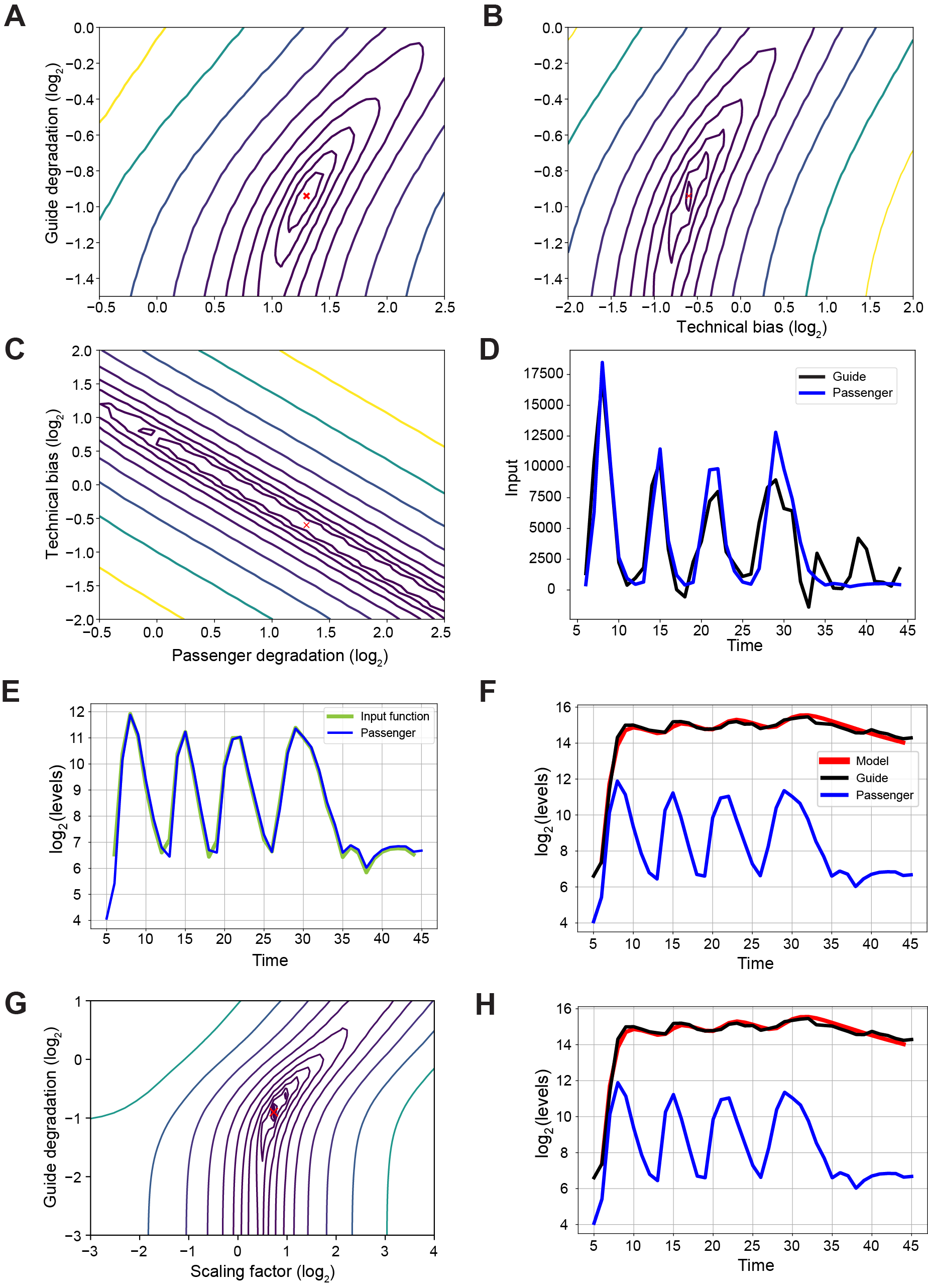
**

**Appendix Figure 2. Comparing full and QSS model on *lin-4*.**

**A)** Contour plot for the error landscape on a $\log_{2}$ scale for both the passenger (X-axis) and guide (Y-axis) degradation rates using the full model, for the minimum value of the other parameters. The red cross represents the estimated minimum.

**B)** Contour plot for the error landscape on a $\log_{2}$ scale for the guide degradation rate (X-axis) and the technical bias (Y-axis) using the full approach, for the minimum value of the other parameters. The red cross represents the estimated minimum.

**C)** Contour plot for the error landscape on a $\log_{2}$ scale for the technical bias (X-axis) and the passenger degradation rate (Y-axis) using the full approach, for the minimum value of the other parameters. The red cross represents the estimated minimum.

**D)** Estimated input functions for the guide (black) and the passenger (blue) strands using the full approach.

**E)** Estimated input function for the passenger strand (green) and passenger strand levels (blue) over time using the full approach in $\log_{2}$ scale.

**F)** Levels over time for the real passenger (blue), real guide (black) and estimated guide (red) strands using the full approach in $\log_{2}$ scale.

**G)** Contour plot for the error landscape on a $\log_{10}$ scale for the scaling factor (X-axis) and guide degradation rate (Y-axis) using the QSS approach, for the minimum value of the other parameters. The red cross represents the estimated minimum.

**H)** Levels over time for the real passenger (blue), real guide (black) and estimated guide (red) strands using the QSS approach in $\log_{10}$ scale.

*Modelling oscillatory degradation*

We explored the implications of oscillatory guide degradation under oscillatory synthesis. To do so, we defined the following model,

$\frac{d\hat{g}}{dt}=f_{\alpha}\left( t \right)-f_{\beta}\left( t \right)\cdot\hat{g}\left( t \right), (S13)$

where $f_{i}(t)$ is a periodic function representing rhythmic synthesis or degradation. If $f_{\beta}$ is non-constant, the analysis of the model becomes much more complicated, even dealing with simple sinusoidal functions. Therefore, we focused on the modelling of miR-235 using this equation to determine whether this could recapitulate the observations.

We chose a non-linear function to represent (possible) pulsatile dynamics, for both synthesis and degradation, by filtering a cosine through a normalised Gaussian function, i.e.

$\begin{matrix} f_{i}\left( t \right)=\sigma_{i}\frac{\exp\left( -\lambda_{i} h_{i}\left( t \right) \right)-exp(-\lambda_{i})}{1-exp(-\lambda_{i})}+\rho_{i}, \\ h_{i}\left( t \right)=1-2\cos\left( \omega t-\phi_{i} \right), \end{matrix} (S14)$

where $\rho$ represents basal production or degradation rate and $\sigma$the maximum value for production or degradation. The phase of the function is represented by $\phi$. Finally, the parameter $\lambda$ represents the decay rate of the Gaussian function and determines how pulse-like the oscillatory function is: for sufficiently low values $\lambda$, $f(t)$ resembles a cosine function, while larger values of $\lambda$ replicates bursts of synthesis or degradation. (These differences can be seen in Appendix Figure 3, the estimated values for the miR-235 fit in Figure 6B.)

The model was fitted to miR-235 guide dynamics in a time-window in which the period remains relatively constant for two oscillations. To ensure a reasonable minimum was found, the fitting processes was performed in several sequential steps via the *scipy* implementation in Python of the Nelder-Mead minimiser (as described in the previous section). Firstly, $f_{\alpha}$ was fitted to the passenger strand levels (in line with the QSS assumption), by minimising the squared difference between $f_{\alpha}$ and $p$. Secondly, the values of $f_{\beta}$ were fitted by integrating the equation using the computed $f_{\alpha}$ and minimising the squared difference between the predicted guide levels and the actual ones.

Finally, using the computed parameters as the starting point, the model was refined by minimising the estimated guide levels, allowing for all the parameters to be changed freely. The minimiser struggled to converge to a final solution, as iterations were exhausted. Nonetheless, all the iterations gave similar fits to the miR-235 model, showing a substantial improvement over the constant degradation model, highlighting the need of periodic degradation to fully reconstruct guide dynamics (Figure 6B). Interestingly, removal of oscillatory degradation led to phase-shifted, damped oscillations, at a higher mean level, which is in line with the miR-235 levels in *ebax-1(tm2321)* worms (Figure 6A,B).

Interestingly, the model exhibited pulsatile synthesis ($\lambda_{\alpha}\approx7$) but seemed to favour a sinusoidal degradation rate ($\lambda_{\beta}\ll0.1$) (Appendix Figure 3). Furthermore, there is a quarter of a cycle phase-shift between degradation and synthesis, in which a stage of large degradation precedes a spike in production and then is followed by a state in which levels remain relatively constant due to the lack of synthesis and degradation.


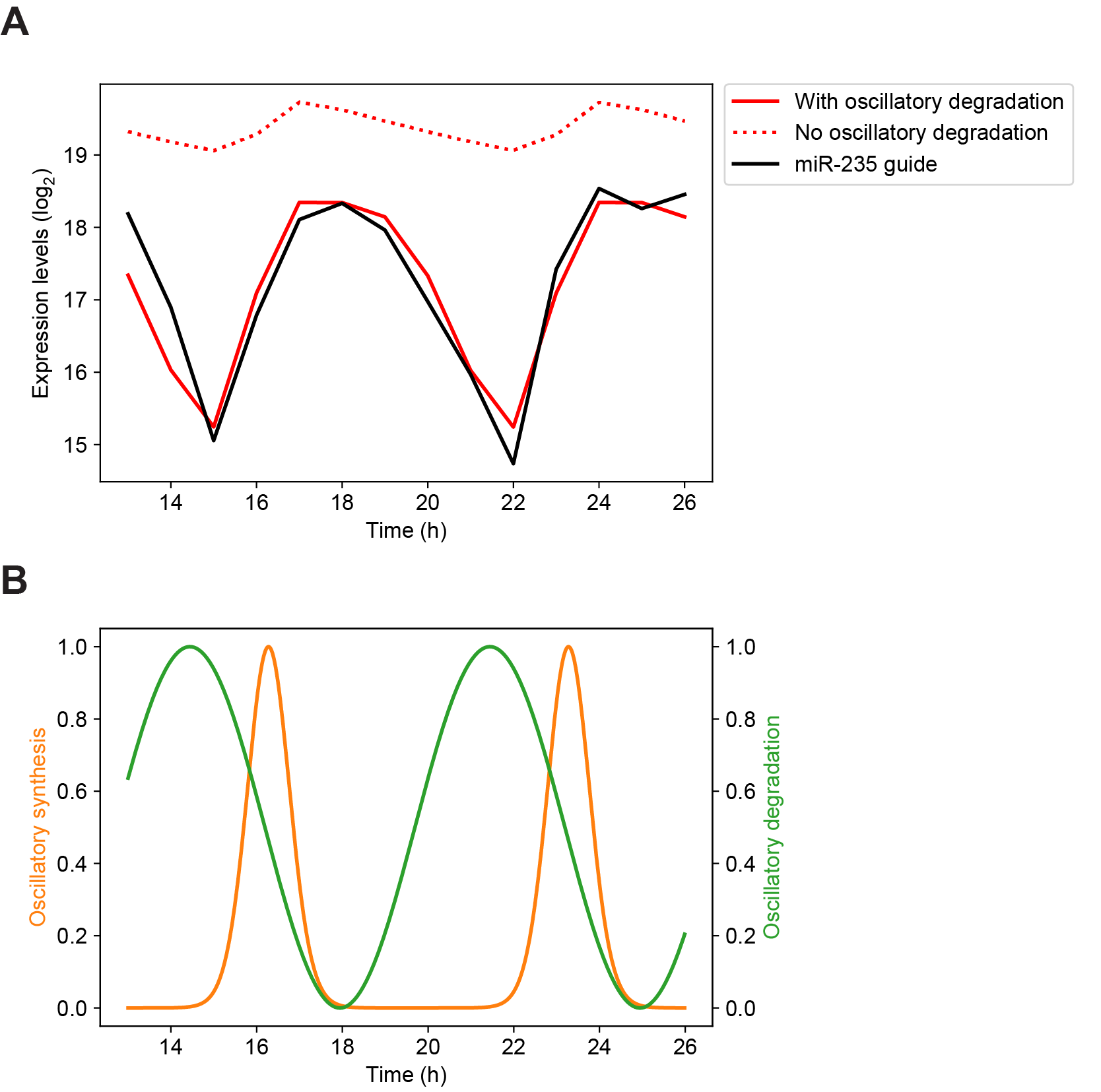


**Appendix Figure 3: Estimated predicted oscillatory synthesis (orange) and degradation (green) over time for miR-235.** Estimated values for the fit shown in Figure 6B are given. The phase shift between both functions is $\Delta\phi=\pi/2$, in which degradation precedes synthesis. Values for $\lambda$ are $\lambda_{synthesis}=0.7$ and $\lambda_{degradation}<0.1$.
