# Supplementary material for: Dynamics of miRNA accumulation during C. elegans larval development
Source: Nucleic Acids Res. 2024 Feb 21;52(9):5336–55. doi: 10.1093/nar/gkae115 (PMC11109986; doi:10.1093/nar/gkae115)
Supplement: gkae115_Supplemental_files [file gkae115_supplemental_files.zip › Supplementary Table Legends.docx]

**Supplementary Table Legends**

**Table S2:** Sequences of synthetic nucleic acids used in this study.

**Table S3:** Time courses sampled and analyzed in this study
