# Supplementary material for: Dynamics of miRNA accumulation during C. elegans larval development
Source: Nucleic Acids Res. 2024 Feb 21;52(9):5336–55. doi: 10.1093/nar/gkae115 (PMC11109986; doi:10.1093/nar/gkae115)
Supplement: gkae115_Supplemental_files [file gkae115_supplemental_files.zip › Supplementary_v6_revised_proofs.pdf]

Supplementary Information

Dynamics of miRNA accumulation during *C. elegans* larval development

Smita Nahar[1,*], Lucas J. Morales Moya[1,*], Jana Brunner[1,2], Gert-Jan Hendriks[1], Benjamin Towbin[1,3], Yannick P. Hauser[1,2], Giovanna Brancati[1,2], Dimos Gaidatzis[1,4,5], Helge Großhans[1,2,5]

[1] Friedrich Miescher Institute for Biomedical Research (FMI), Basel, Switzerland
[2] University of Basel, Basel, Switzerland.
[3] University of Bern, Bern, Switzerland
[4] SIB Swiss Institute of Bioinformatics, Basel, Switzerland
[5] Correspondence to: helge.grosshans@fmi.ch or dimosthenis.gaidatzis@fmi.ch
[*] Co-first authors

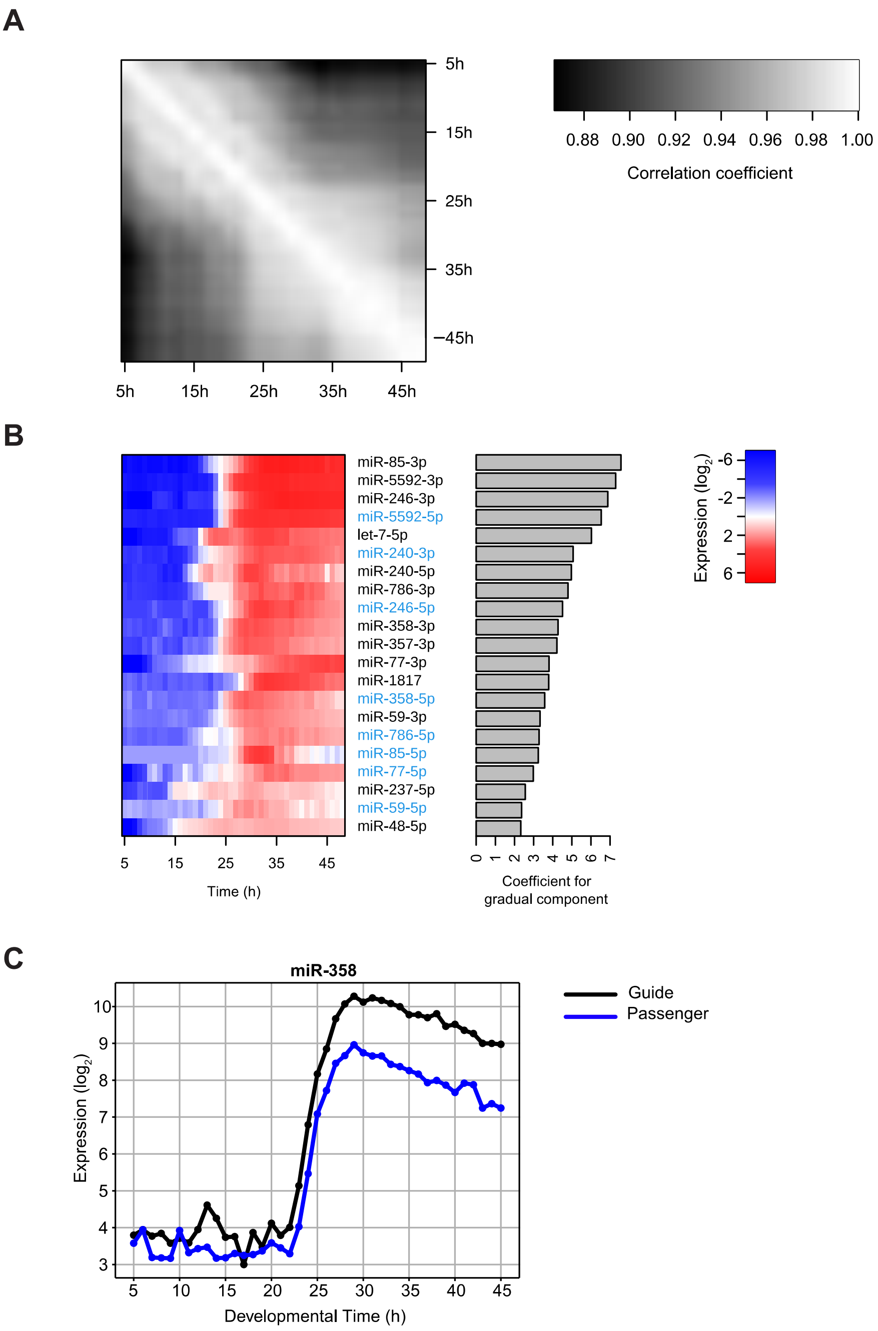

Figure S1. A) Pairwise correlation plot of log_2-transformed count data of all miRNAs sequenced from 5-48 h at 25°C. The color key visualizes the values from low correlation (black) to high correlation (white). The bright off-diagonals reveal the presence of a periodic expression signal, whereas the progressive decrease in correlation most noticeable after ~25 h indicates a gradually changing expression signal.

B) A subset of miRNAs in the *gradual* cluster increases during the L3 stage, at ~25 h. The heatmap, showing mean-normalized, log_2-transformed expression data, was sorted by the coefficients of the gradual component. Passenger strands are labelled in *light blue*, guide strands in *black.*

C) Log_2-transformed expression of miR-358 guide (black) and passenger (blue) strands of the gradual cluster from 5-48 h at 25°C obtained by small-RNA sequencing illustrates massive expression changes around 25 h.

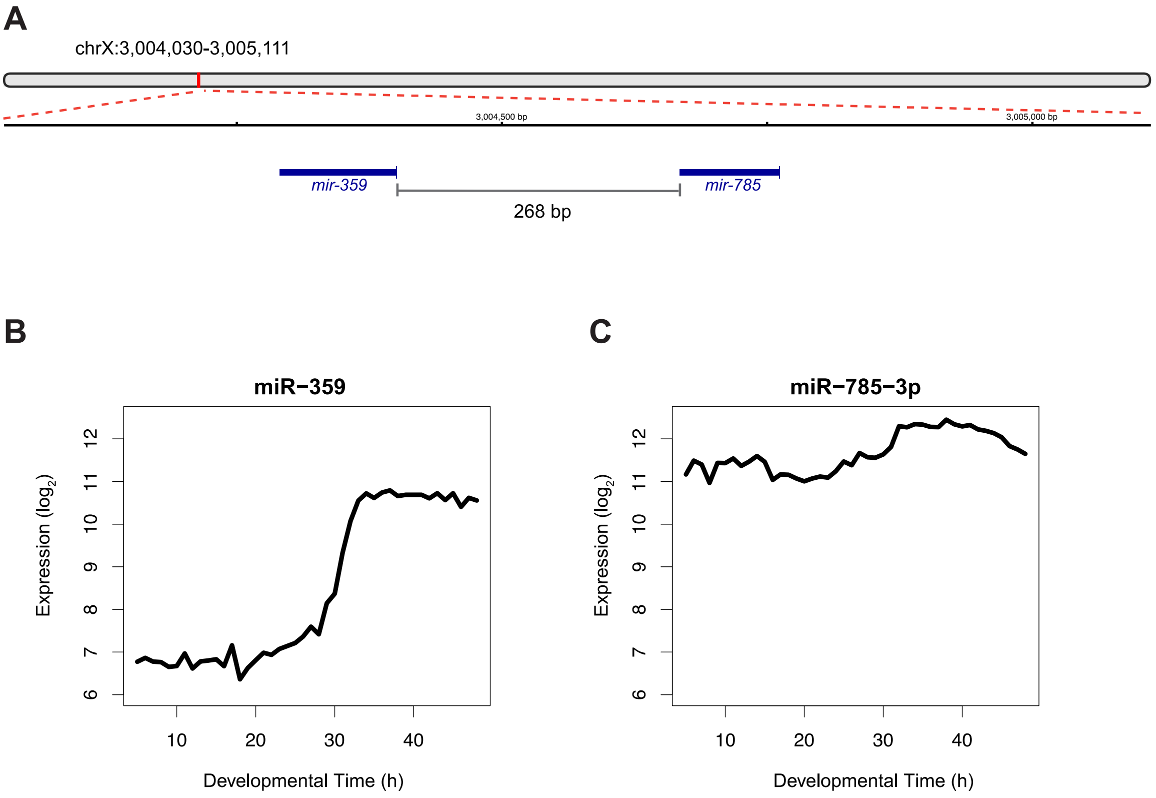

Figure S2. A) Genome browser view of the *mir-359~mir-785* locus. Although closely clustered, consistent with co-transcription, the expression patterns of B) miR-359 and C) miR-785-3p (guide), measured by small-RNA sequencing, differ greatly.

A

$$\frac{dx}{dt} = \sin\left(\frac{2\pi}{6.8}t\right) - \beta x$$

B

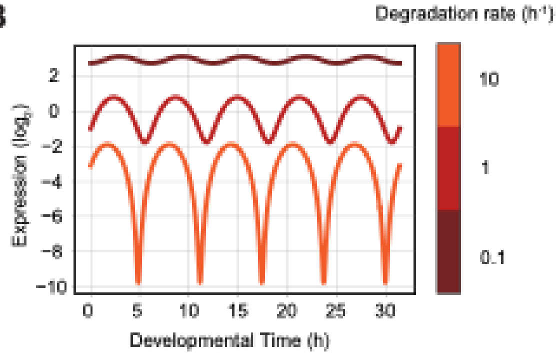

C

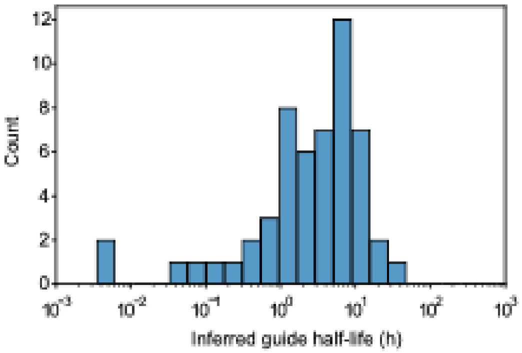

Figure S3. Increasing degradation rates increase the relative amplitude of a signal as well as decreasing average levels. A) Equation simulating RNA levels x under oscillatory gene expression with amplitude 1 and period of 6.8 h and degradation rate β. B) Expression levels over time for different degradation rates (0.1, 1, 10 h^{-1}). C) Histogram of half-lives for estimated guide strands. Numerical values can be computed from Supplementary Table S1 as $\tau_{1/2} = \ln(2)/\beta_g$. miRNAs (n = 2) with half-lives longer than 100 hours are excluded.

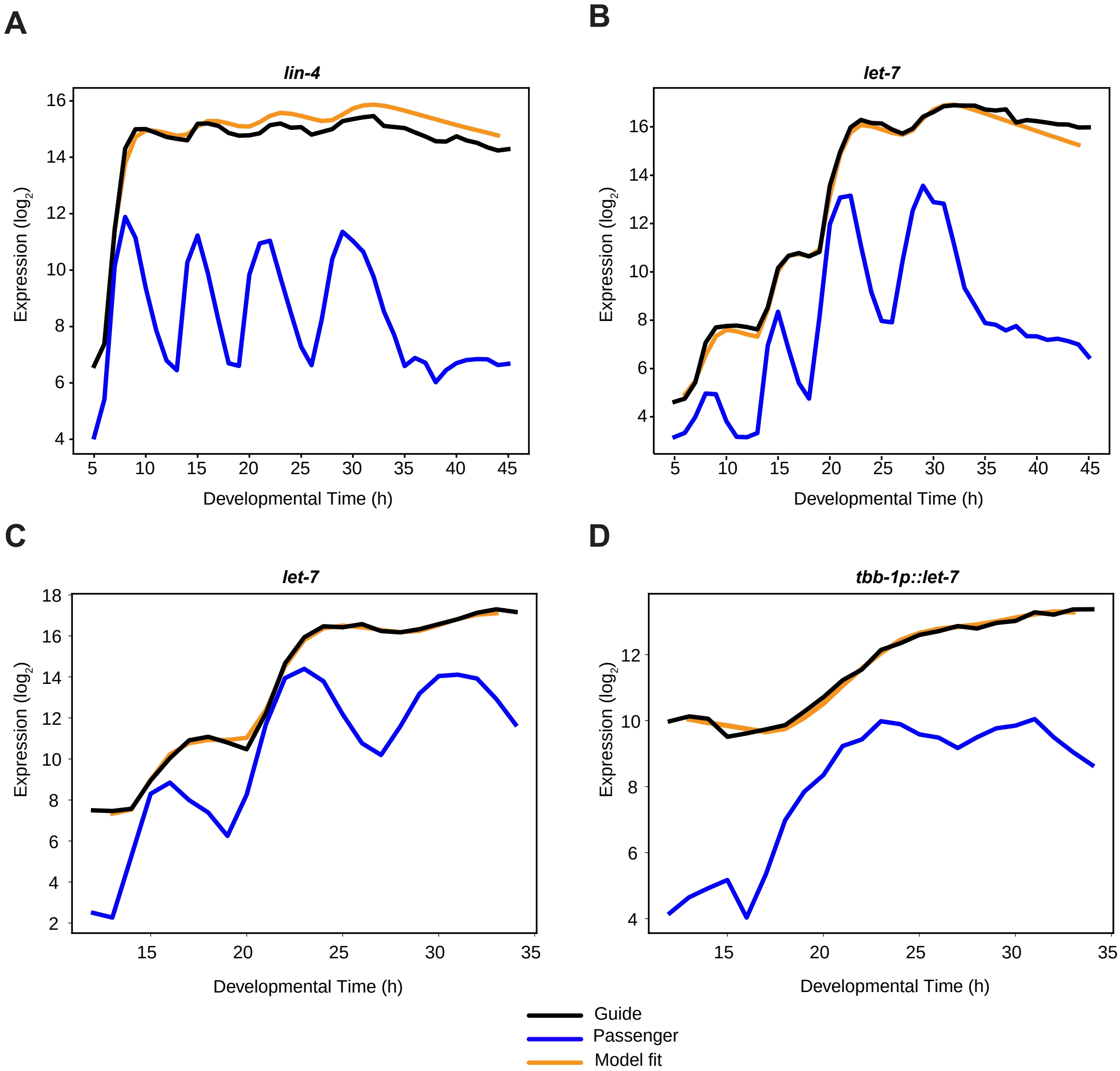

Figure S4. Modelling of A) *lin-4* and B) *let-7* guide strand dynamics using the estimated *let-7* (A) and *lin-4* (B) half-lives, respectively. Differences between guide strand dynamics are not a consequence of different half-lives, as patterns of the model fit (orange) remain qualitatively similar to the guide strand patterns (black) despite the swap of half-lives. Modelling of *let*-7 guide strand dynamics using the passenger strand in C) wild-type conditions and D) under the *tbb-1* promoter in RT-qPCR time course collected hourly from 12-34 h at 25°C. Both C) and D) gave identical values for β_g ($0.127\ h^{-1}$, corresponding to $\tau_{½} = 5.5\ h$), suggesting that differences arise from different passenger dynamics.

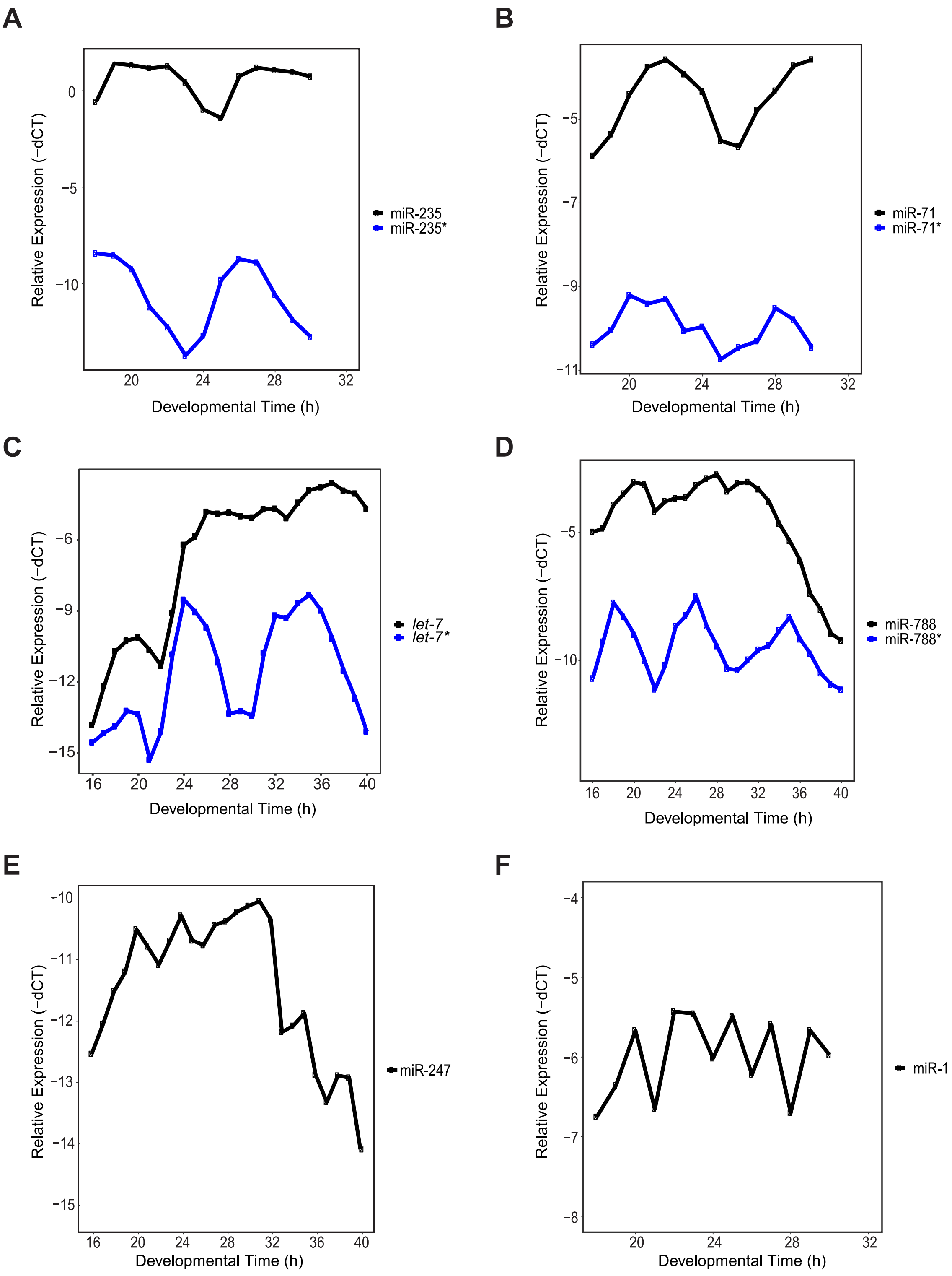

Figure S5. Independent RT-qPCR time courses were sampled hourly at 25°C to measure the levels of the indicated different miRNAs by Taqman assays. A) miR-235, B) miR-71 and F) miR-1 were measured in TC6 (sampled from 18-30 h). C) *let-7*, D) miR-788 and E) miR-247 were measured in TC8 (16-40 h). The Ct values of the respective miRNAs were normalized to the control, sn2841 RNA, by the following equation: –dCT = − (miRNA CT values – sn2841 CT values). miR-1 (F) is included as an example of a miRNA with largely invariant expression.

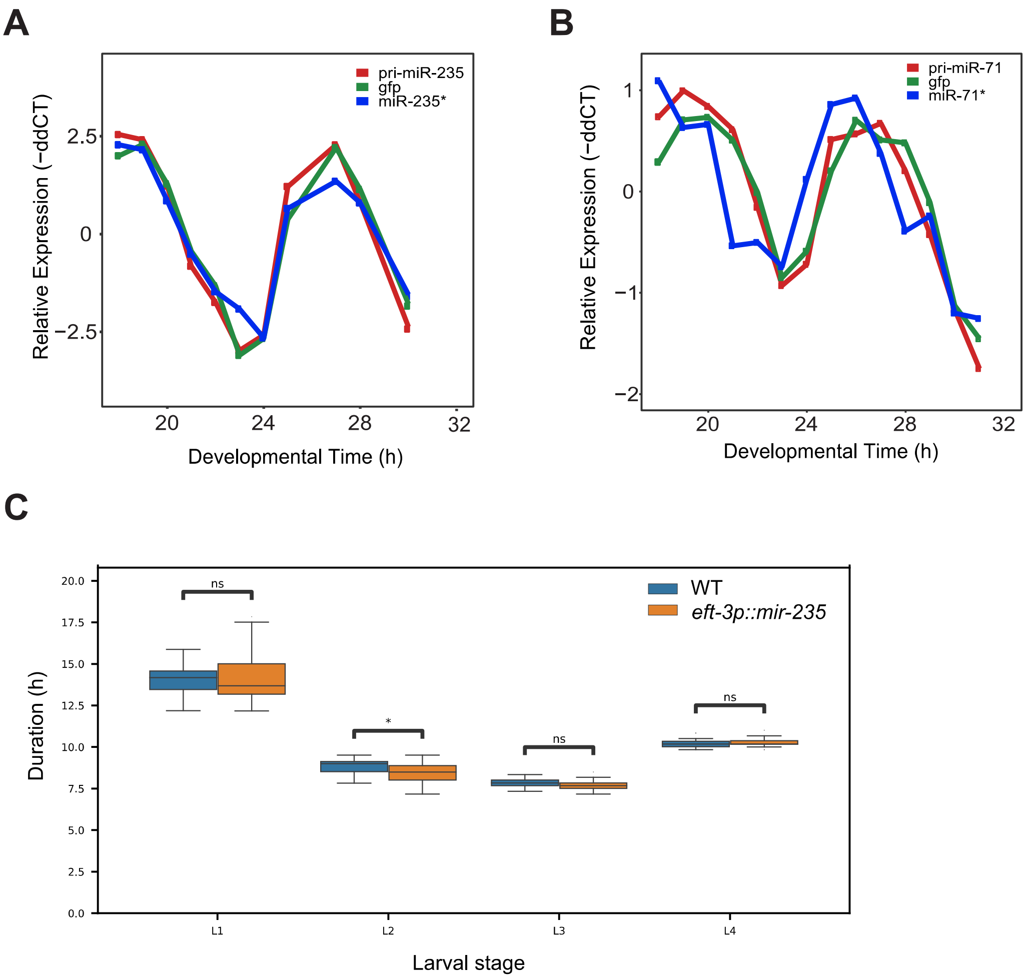

Figure S6. A)-B) Levels of endogenous primary miRNA transcript, passenger strand, and *gfp* mRNA produced from the promoter of *mir-235* (A) and *mir-71* (B). Quantification by RT-qPCR was performed on the same RNA samples for all three RNA species, collected for the strain HW2280 (A, time course TC2) and for the strain HW2770 (B, TC3), respectively. Strains were grown at 25°C and sampled hourly at the indicated timepoints. Both *gfp* transcripts recapitulate endogenous pri-miRNA oscillations, indicating that the promoter is sufficient to drive oscillations of primary transcript and passenger miRNAs.

C) Quantification of larval stage duration in WT and eft-3p-*mir-235* by luciferase assay. No obvious change in developmental synchrony and developmental speed was observed. Significance was calculated by Mann Whitney U test (* p<0.05).

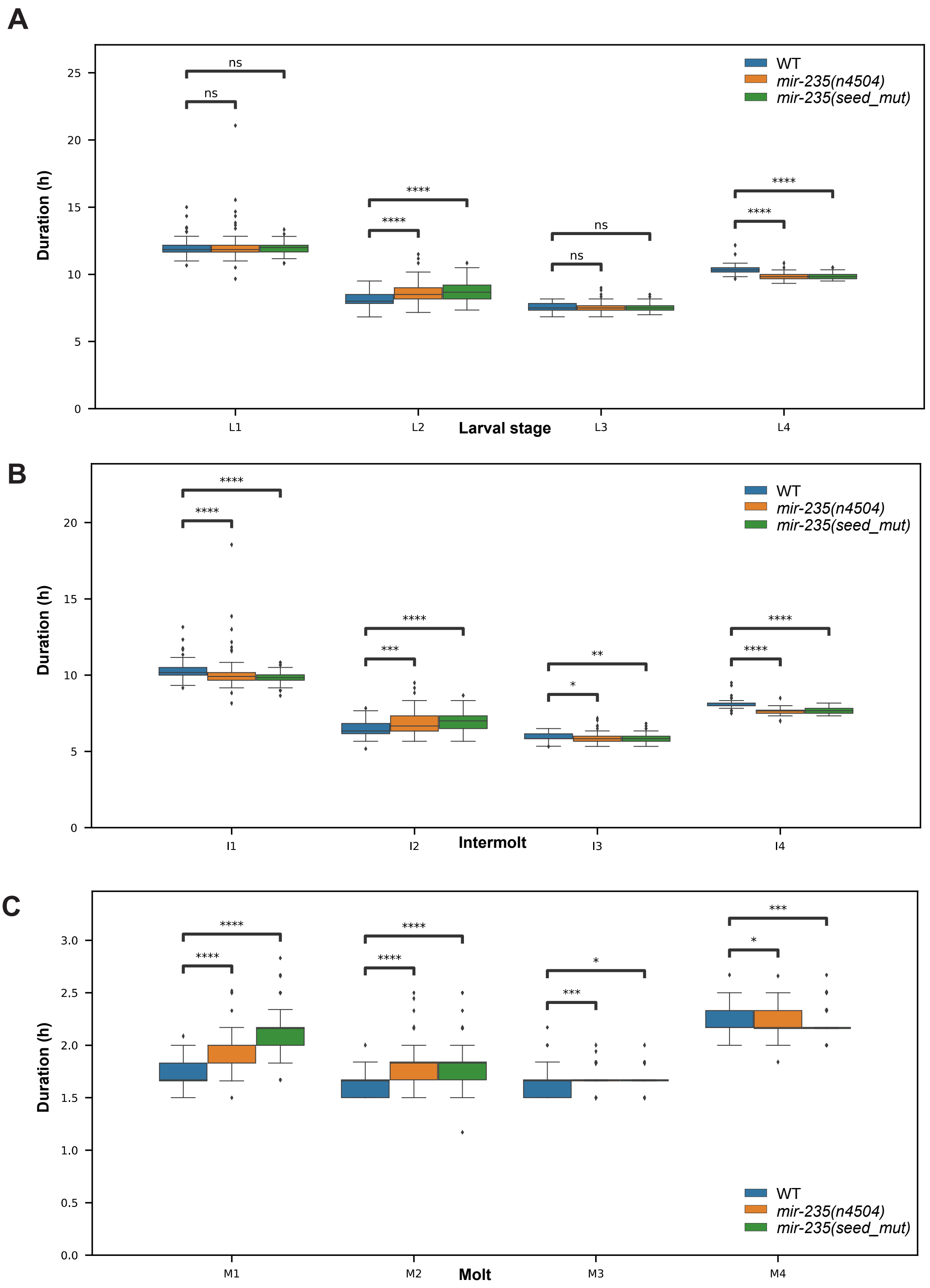

Figure S7. Quantification of A) larval stage B) intermolt and C) molt durations in wild-type (WT), *mir-235(n4504)* and *mir-235_seed mut* animals by luciferase assay. No obvious changes in developmental synchrony were observed, and changes in developmental tempo were minor. Significance was calculated by Mann Whitney U test (* $p<0.05$, ** $p<0.01$, *** $p<0.001$, **** $p<0.0001$).

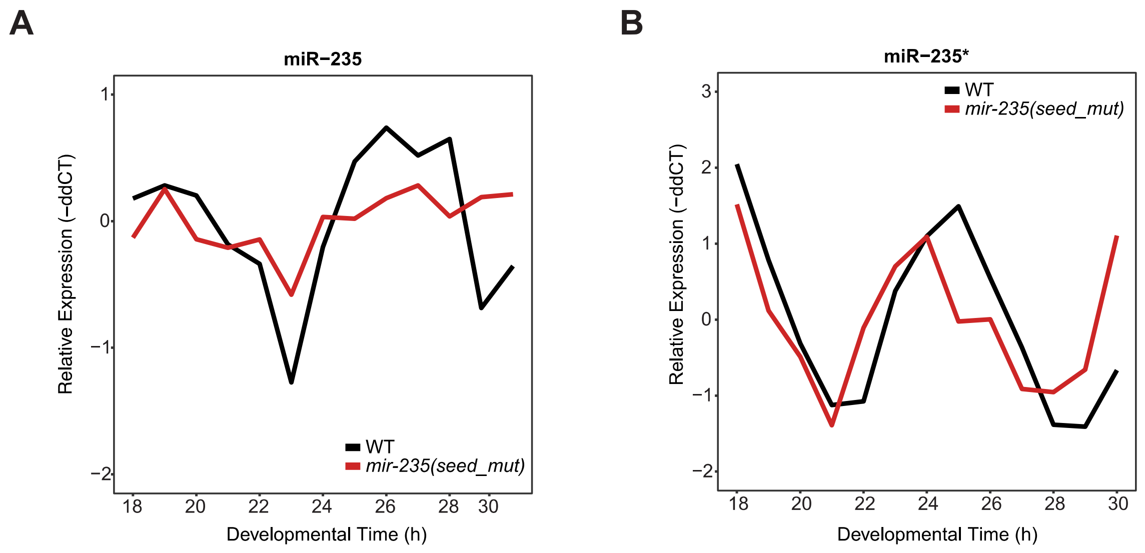

Figure S8. RT-qPCR validation of A) miR-235 and B) miR-235* in WT and *mir-235(seed_mut)* worms sampled hourly from 18-30 h at 25°C. Reactions of RT-qPCR were performed in triplicates, the values were averaged and normalized to the reference gene, by following equation: –dCT = − (miRNA Ct values – reference Ct values). sn2841 RNA was used as internal control for miRNA The following equation was used to quantitate the relative expression shown in the y-axis: -ddCT = - (dCT – dCTmean).

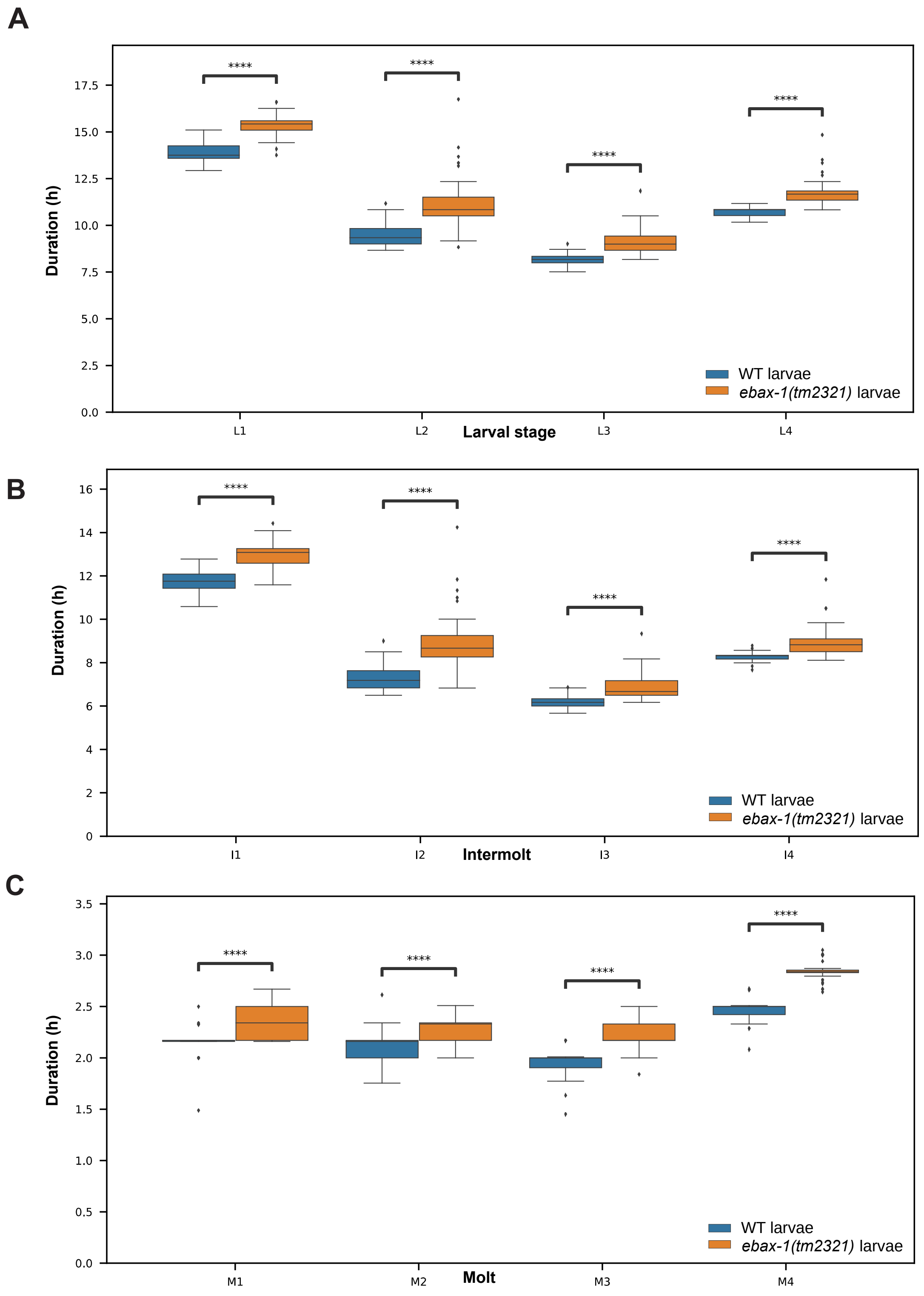

Figure S9. Quantification of A) intermolt B) molt and C) larval stage durations in WT and *ebax-1(tm2321)* larvae. Synchronized L1 larvae were examined by a luciferase assay to quantify the developmental durations. The *ebax-1(tm2321)* mutants showed extended larval development for all the larval stages. Significance was calculated by Mann Whitney U test ((* p<0.05, **** p<0.0001).

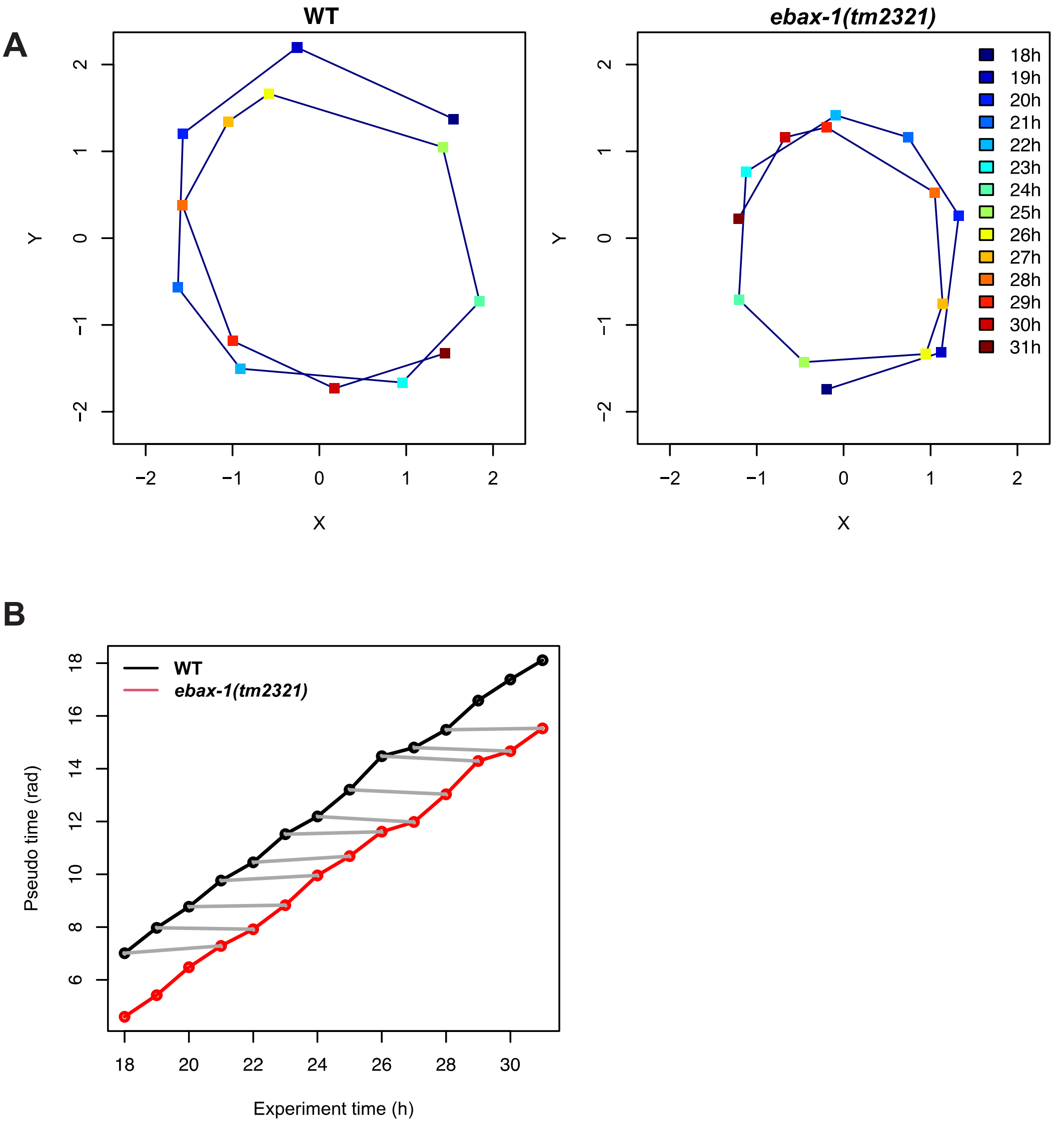

Figure S10. A) Scatter plot visualizing the extent of oscillations in the N2 and *ebax-1(tm2321)* mutant time course data. Based on prior phase information of 3,739 genes that oscillate during *C. elegans* larval development (1), we calculated the average expression of 4 gene sets (0±45, 90±45, 180±45 and 270±45 degrees) and subtracted the two anti-phase pairs 0:180 and 90:270 to obtain two orthogonal expression readouts X and Y (see Methods), which are depicted in the scatter plot. Synchronized worms during larval development display circular trajectories that progress in a counterclockwise direction.
B) Scatter plot comparing the experimental time (x-axis) to the inferred pseudo time (y-axis) for N2 (black line) and the *ebax-1(tm2321)* mutants (red line). The gray lines connect the pairs of N2 and the *ebax-1(tm2321)* mutants with a roughly similar pseudo time. The timepoints of 18-28 h in the wild-type with respect to timing are roughly comparable to 21-31 h in the *ebax-1(tm2321)* mutants.

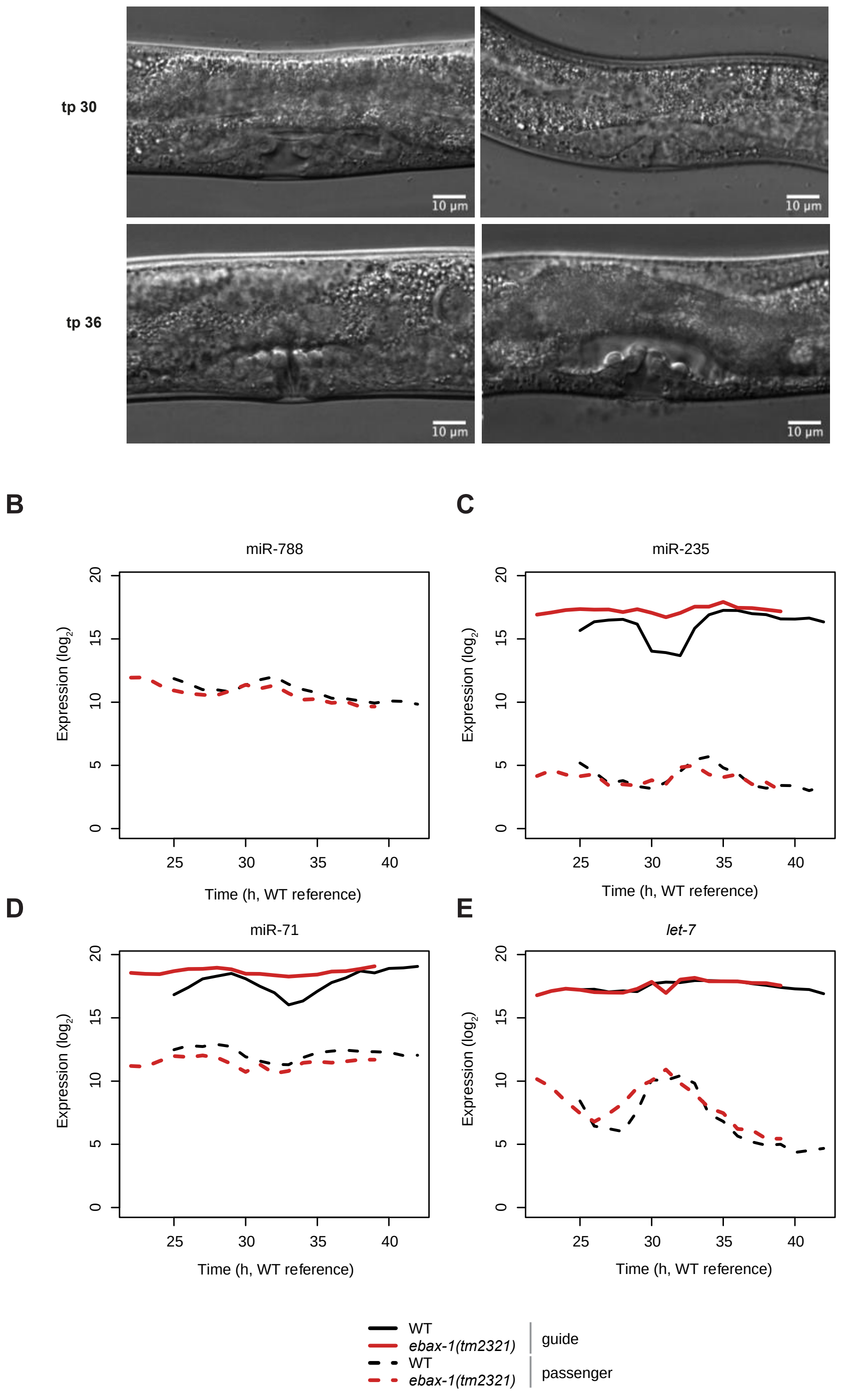

Figure S11. A) Micrographs of WT and *ebax-1(tm2321)* mutant vulvae in TC9 (Fig. 7). At timepoint (tp) 30, vulval morphology corresponds to L4.4 in WT, and L4.1 in *ebax-1(tm2321)* mutant animals. At tp 36, the WT vulva is at >L4.9, the *ebax-1(tm2321)* mutant vulva at L4.7. These data are consistent with a ~ 3 h-delay between the wild-type and the mutant animals. B)-E) Expression of miRNAs revealed by small RNA sequencing of WT and *ebax-1(tm2321)* mutant animals from TC9. Synchronized L1 larvae were plated on food at 25°C and sampled hourly between 25 h and 42 hour after plating. The time of *ebax-1* mutant samples is adjusted by -3 h to compensate for the developmental delay relative to WT.

	Strand	μ $\left(\frac{au}{h^{-1}}\right)$	β_g (h^{-1})	C (au)
miR-239a	5p	0.35	<0.01	1703.90
miR-250	3p	0.79	<0.01	17539.70
miR-77	3p	1.24	0.02	22.51
miR-51	5p	3.19	0.04	103841.65
miR-49	3p	0.00	0.04	7903.26
miR-52	5p	0.19	0.05	176831.02
miR-244	5p	<0.01	0.05	184560.62
miR-2	3p	1.26	0.05	69738.23
miR-57	5p	316.23	0.06	1066567.48
miR-124	3p	1.46	0.06	2085.69
miR-1022	5p	3.98	0.07	7251.44
miR-232	3p	1.80	0.07	4601.07
miR-791	5p	0.05	0.08	410.88
let-7	5p	3.54	0.08	22.34
miR-255	3p	0.37	0.08	146.12
miR-84	5p	1.73	0.09	5472.22
miR-1824	5p	0.13	0.09	127.18
miR-44	3p	31.62	0.09	510368.86
miR-4813	3p	0.68	0.10	2834.50
miR-45	3p	5.28	0.10	283233.00
miR-70	3p	79.43	0.11	36718.78
lin-4	5p	4.85	0.11	167.25
miR-228	5p	39.84	0.12	1896279.80
miR-237	5p	35.17	0.13	9699.30
miR-238	3p	19.01	0.14	37959.07
miR-235	3p	316.23	0.14	85074.00
miR-90	3p	199.53	0.16	566718.51
miR-253	3p	13.29	0.16	3730.97

	Strand	μ $\left(\frac{au}{h^{-1}}\right)$	β_g (h^{-1})	C (au)
miR-795	5p	0.79	0.17	3163.56
miR-230	5p	0.23	0.17	10700.14
miR-48	5p	13.21	0.17	477.32
miR-40	3p	501.19	0.26	22859.43
miR-59	3p	158.49	0.31	1662.77
miR-231	3p	3.13	0.35	4552.55
miR-41	3p	0.39	0.35	35.17
miR-43	3p	1.92	0.39	2325.10
miR-241	5p	1.99	0.40	53.57
miR-36	3p	19.21	0.42	4489.03
miR-790	5p	0.45	0.49	2609.86
miR-37	3p	18.40	0.50	4301.55
miR-42	3p	1.39	0.54	6431.36
miR-39	3p	12.78	0.55	13242.99
miR-1829b	5p	63.10	0.55	3107.66
miR-2953	5p	0.56	0.58	11.69
miR-1832a	5p	3.67	0.62	164.09
miR-35	3p	595.39	0.90	12215.73
miR-71	5p	5.04	1.22	178726.25
miR-1829c	5p	501.19	1.28	2895.67
miR-784	3p	5.88	1.32	31.95
miR-358	3p	5.33	1.75	6.89
miR-5592	3p	12.95	3.03	7.23
miR-1830	3p	13.06	4.48	187.36
miR-788	5p	90.86	8.01	2441.04
miR-797	3p	32.03	16.56	45.33
miR-240	5p	564.38	194.10	93.83
miR-247	3p	4882.12	198.93	38.34

Table S1. Computed parameter values for different miRNAs using the quasi-steady approach. Values for the scaling factor μ, the guide degradation rate β_g and the initial condition C are shown for each of the miRNAs we modelled.

References

1. Meeuse, M.W., Hauser, Y.P., Morales Moya, L.J., Hendriks, G.J., Eglinger, J., Bogaarts, G., Tsiairis, C. and Grosshans, H. (2020) Developmental function and state transitions of a gene expression oscillator in Caenorhabditis elegans. *Mol Syst Biol*, **16**, e9975.
